# Evaluation of the First Year(s) of Physicians Collaboration on an Interdisciplinary Electronic Consultation Platform in the Netherlands: Mixed Methods Observational Study

**DOI:** 10.2196/33630

**Published:** 2022-04-01

**Authors:** Sanne M Sanavro, Henk van der Worp, Danielle Jansen, Paul Koning, Marco H Blanker, Prisma Platform Study Group

**Affiliations:** 1 Department of General Practice and Elderly Care Medicine University of Groningen Groningen Netherlands; 2 Prisma Siilo Holding BV Amsterdam Netherlands; 3 See Acknowledgments

**Keywords:** primary care, digital consultation, interdisciplinary, specialist care

## Abstract

**Background:**

Complexity of health problems and aging of the population create an ongoing burden on the health care system with the general practitioner (GP) being the gatekeeper in primary care. In GPs daily practice, collaboration with specialists and exchange of knowledge from the secondary care play a crucial role in this system. Communication between primary and secondary care has shortcomings for health care workers that want to practice sustainable patient-centered health care. Therefore, a new digital interactive platform was developed: Prisma.

**Objective:**

This study aims to describe the development of a digital consultation platform (Prisma) to connect GPs with hospital specialists via the Siilo application and to evaluate the first year of use, including consultations, topic diversity, and number of participating physicians.

**Methods:**

We conducted a mixed methods observational study, analyzing qualitative and quantitative data for cases posted on the platform between June 2018 and May 2020. Any GP can post questions to an interdisciplinary group of secondary care specialists, with the platform designed to facilitate discussion and knowledge exchange for all users.

**Results:**

In total, 3674 cases were posted by 424 GPs across 16 specialisms. Most questions and answers concerned diagnosis, nonmedical treatment, and medication. Mean response time was 76 minutes (range 44-252). An average of 3 users engaged with each case (up to 7 specialists). Almost half of the internal medicine cases received responses from at least two specialisms in secondary care, contrasting with about one-fifth for dermatology. Of note, the growth in consultations was steepest for dermatology.

**Conclusions:**

Digital consultations offer the possibility for GPs to receive quick responses when seeking advice. The interdisciplinary approach of Prisma creates opportunities for digital patient-centered networking.

## Introduction

In the Dutch health care system, general practitioners (GPs) have a coordinating role as generalists, functioning as gatekeepers to secondary care. This model requires that patients initially consult a GP who provides expert generalist medical care for their health care problem and considers the need for referral to more specialist care.

Unfortunately, pressures on the health care system have increased due to the growth in both the chronicity and the complexity of health problems [1,2]. Although GPs care for over 95% of medical problems that present during consultations, referral to secondary care has also increased, resulting in greater health care costs and growing waiting lists [3,4].These issues can be addressed by providing GPs with closer support from secondary care, assuming there are effective routes for knowledge exchange [5-9]. However, the most commonly used tools for communication between primary and secondary care have important shortcomings. For example, GPs and hospital specialists are often mutually unavailable at the same time, meaning that telephone conversations can be interruptive. Whereas e-consultations may solve the problem of asynchronous availability, they are limited by being monodisciplinary, one-on-one, and mostly noninteractive [6,10-13]. Digital response times may also vary by specialism. By contrast, team-based case collaboration on a patient-centered network of health care professionals could facilitate communication and knowledge transfer [14-16]. The secure Siilo app offers a useful platform to host such a service [17,18].

In this study, we describe the development of the Dutch Prisma platform within the secure Siilo app and evaluate the usage and consultations in the first 2 years since its introduction, including the diversity of topics and number of physicians involved.

## Methods

### Study Design

We performed a retrospective mixed methods study using quantitative information from the Prisma platform and a qualitative evaluation of consecutive cases posted on the platform from its inception in July 2018 to May 2020.

### The Prisma Platform

The Prisma platform initially facilitated digital interprofessional consultation for patients with orthopedic problems, but more recently, it has expanded to include other specialties. GPs with full access to the closed digital environment of the platform generate cases by providing anonymized patient information with a question. All GPs and specialist users are connected in so-called tiles by specialty (eg, orthopedics, internal medicine, palliative care) to facilitate engagement by consultants with complementary expertise (eg, rheumatologists, orthopedic surgeons, sports medicine physicians, and radiologists participate via the orthopedics tile). All users can engage with each tile and upload attachments or links to relevant information, such as laboratory results, pictures, or guidelines. The main language used on the platform is Dutch.

Two GP groups are active on this platform: 1 with full access (able to generate cases and respond to others) and 1 with a read-only account. Specialists participated voluntarily; separate from their hospital work and without reimbursement for their activity on the platform. Because they were not reimbursed, the number of GPs was limited during the development phase to avoid overloading the specialists. All users, both GPs and specialists, were located in various regions of the Netherlands. Specialists preferably respond within 24-48 hours by answering questions, seeking more information, or engaging in discussion. All GPs with access to the platform can read and respond to posted cases. In this way, the platform allows for a dynamic exchange of information and learning to support the GP in daily practice. Throughout the process, the GP remains responsible for the care provided to the patient and will decide, in consultation with the patient, how to proceed with further treatment.

### Data Collection

A data analyst at Siilo provided pseudonymized details for all consecutive cases, replacing usernames with a job title and a number (eg, GP-1, GP-2, neurologist-1). Each post was summarized as a user code, timestamp, and verbatim transcript, and these were grouped by case for each tile. Data were analyzed qualitatively and quantitatively. As we performed a retrospective descriptive study, we did not predefine our sample size.

### Qualitative Analysis

Text files were imported into the Atlas.ti program [19] for qualitative assessment by a research group comprising 20 senior medical students (coders) supervised by an internist (SS), a medical sociologist (DJ), a GP epidemiologist (MB), and a senior researcher (HW). The Prisma affiliate (PK) was not involved in this phase.

We used a predefined coding tree to structure the qualitative assessment (Multimedia Appendix 1). Before applying this to all cases, a random sample of 10 cases was initially coded by all coders. The results of this preliminary coding were then checked in pairs and discussed in 5 subgroups with 2 supervisors. Coders were actively invited to discuss the applicability of codes and to add new codes if needed. After this, coders were grouped by tile and at least 50 cases per tile were coded in duplicate with mutual blinding. This was followed by group discussion in consensus meetings per subgroup, after which the remaining cases were coded.

The coding tree comprised the following: basic patient characteristics, such as age, gender, and comorbidity; the topic of the question; and both the type of question and the type of answer (eg, diagnostic, therapeutic, or referral for both). Codes for symptoms and diseases followed the International Classification in Primary Care (ICPC) [20], with multiple codes permitted.

### Quantitative Analysis

All codes were imported into IBM SPSS (IBM Corp.) for quantitative analysis. We merged the 16 tiles into 5 categories based on similarities and group sizes: “internal medicine” included internal medicine, infectious disease, palliative care, and medically unexplained physical symptoms; “observation” included gastroenterology, neurology, pulmonology, rheumatology, and cardiology; “surgical” included orthopedics, urology, traumatology, and ear, nose, and throat disease; “female/child” included gynecology and pediatrics; and “dermatology” as a single category. The tile for psychiatry was analyzed and published separately and is therefore excluded from this analysis [21].

An overview of activity on the platform is displayed by plotting the number of GPs (active users and read-only accounts) and the number of cases against time. We estimated the number of users, number of specialisms, number of specialists, and the response time for each case based on user codes and timestamps, and we analyzed the code frequencies for age, gender, case topic (based on the ICPC code), question type, and answer type for each category. Descriptive data were presented as percentages of all cases or as means and SDs. Finally, we used a Sankey diagram to show the linkage between questions and answers.

## Results

### Descriptive Data

The data set started with 25,954 messages for 4013 cases; of these, 1872 messages were excluded for 339 cases. First, we excluded 292 cases because of data extraction errors (n=34), small size, and difficulty to categorize within groups (geriatrics, n=5; ophthalmology, n=40) and because they were already analyzed in a separate study (psychiatry, n=213) [21]. Next, we divided the data within the research team and analyzed the 3721 cases. We excluded another 47 cases because of wrong tile placement (n=19), double case placement (n=10), technical errors (n=8), not coded (n=7), withdrawal by GP (n=2), missing (n=1) (Multimedia Appendix 2). The 3674 included cases were posted by 424 different GPs (median 9 cases per GP), for whom 97 (22.9%) first posts were in response to another case and 327 (77.1%) posts were for new cases.

**Figure 1:**
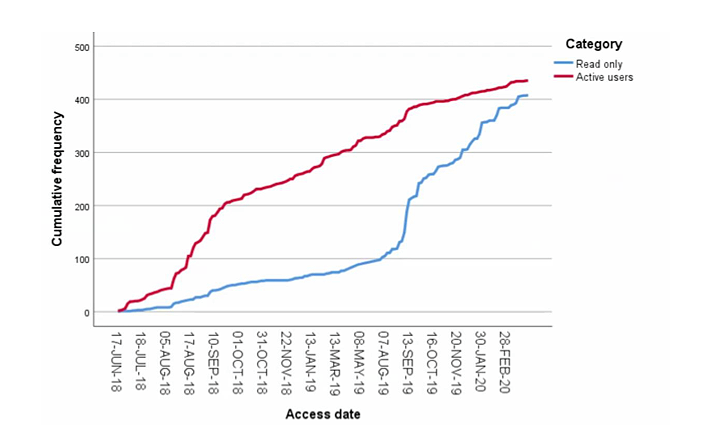
Platform use; number of active and read-only GPs on the platform. GP: general practitioner.

**Figure 2:**
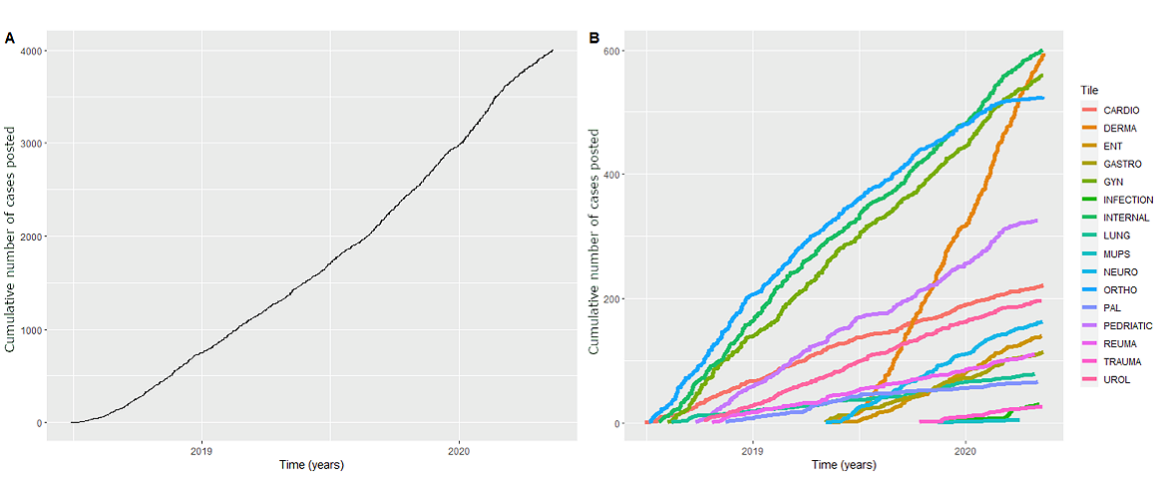
Overall cases of network activity and network activity by tile category. ENT: ear, nose, throat; GYN: gynaecology; MUPS: medically unexplained physical symptoms; PAL: palliative care; UROL: urology.

**Figure 3:**
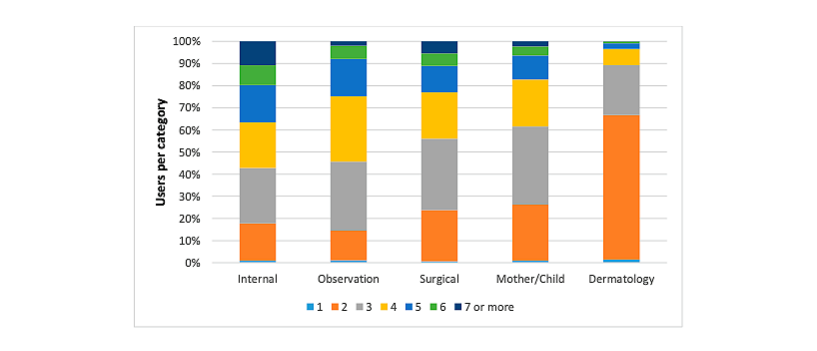
Number of users involved per case. Data are illustrated in 5 tile categories.

**Figure 4:**
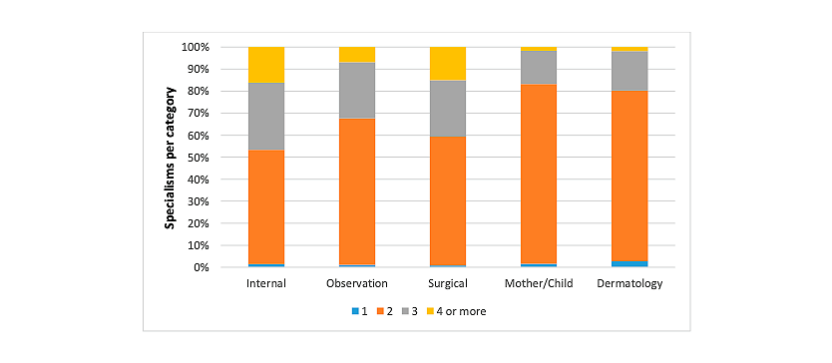
Number of specialisms involved per case. Data are illustrated in 5 tile categories.

Growth of the Prisma platform over time is shown as the number of GPs (active users and read-only accounts; Figure 1), the total number of cases, and the number of cases per tile (Figure 2). The number of cases per category was 677 for internal, 674 for observation, 860 for surgical, 875 for female/child, and 588 for dermatology. Figures 3 and 4 show the number of specialists and specialisms involved per tile category, respectively. For all categories, except dermatology (196/588, 33.3%), most cases included more than 2 users per case. For the internal, observation, and surgical categories, 3 or more specialisms were involved per case in 46.6% (317/680), 32.3% (217/672), and 40.7% (350/860), respectively. In the internal and observation categories, 4 or more health care professionals were engaged per case in 57.2% (389/680) and 54.0% (363/672), respectively.

Case characteristics are presented in Multimedia Appendix 3. No answer was given for 35 cases, with the median time to first response being 76 minutes (IQR 17-320) for the other cases. The shortest response time was seen in the surgery category (median 44 minutes) and the longest was in the dermatology tile (median 252 minutes). Overall, 3508/3674 (95.48%) cases contained specific patient information or patient-specific questions, with the remaining 166 (4.52%) cases including questions that were not specific to the patient. Slightly more than half of all queries concerned females (1948/3674, 53.02%), except for those in the surgical tile where there was a slight male majority (437/860, 50.8%). GPs did not report gender in 8.92% (313/3508) of the patient-specific cases. They also posted a question about more than 1 patient in 4 cases (eg, family members or several patients with the same complaint). Patient age ranged from newborn to 101 years (mean 39.9 years) and the mean age differed by tile category. The GP did not report age for 701 cases.

Topics discussed covered the full range of ICPC codes (Multimedia Appendix 4). The 3 main topics by ICPC code were in the skin, musculoskeletal, and general symptom domains.

### Type of Questions and Answers

Among the 3674 cases, we identified 6691 different questions (mean 1.8 per case) and 10,922 answers (mean 3.03 per case). Multimedia Appendix 5 shows the type of question and answers posted.

Questions concerned (differential) diagnosis in 50.90% (1870/3674), appropriate nondrug treatment in 33.15% (1218/3674), and drug treatment in 27.60% (1014/3674). It was notable that the focus of questions differed between tile categories. Most concerned diagnosis in the internal (358/677, 52.9%), observatory (361/674, 53.6%), and dermatology (424/588, 72.1%) categories; most concerned treatment in the surgical category (431/860, 50.1%); and most concerned medication in the female/child category (378/875, 43.2%).

**Figure 5:**
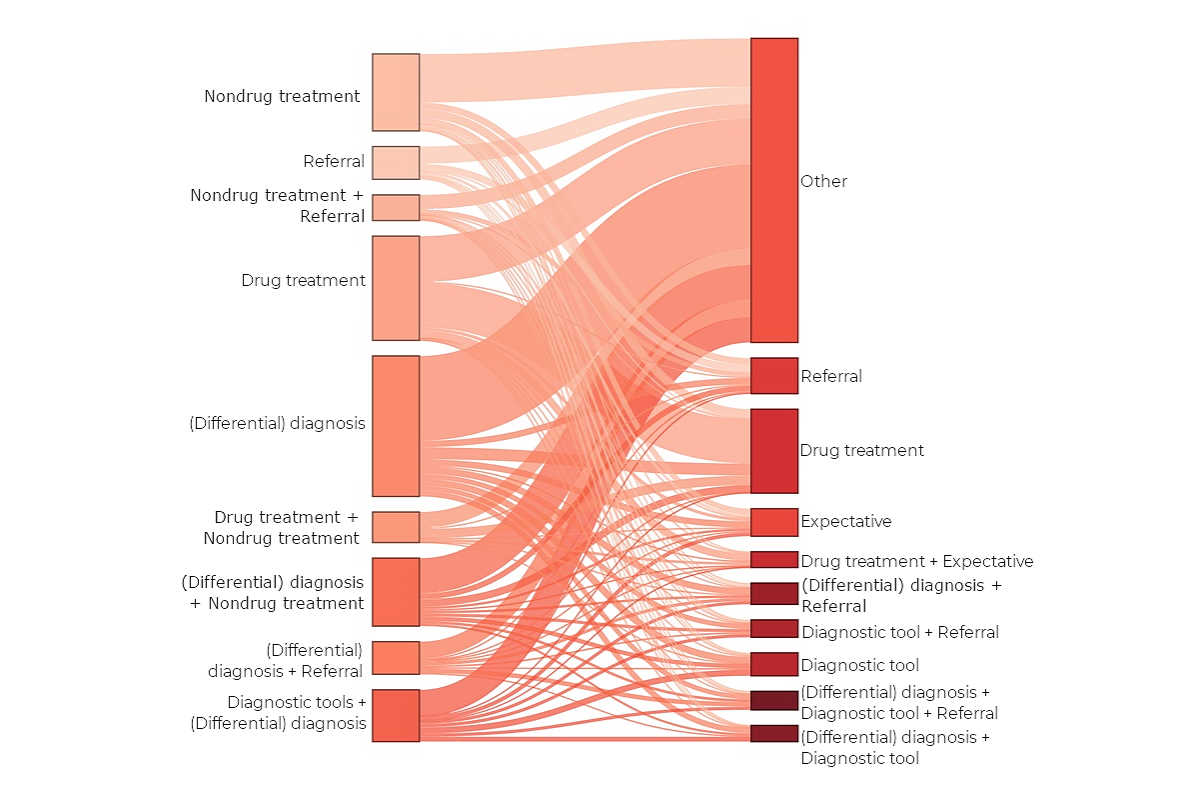
Sankey diagram of dynamics from questions to answers, 9 largest groups.

The Sankey diagram in Figure 5 illustrates the dynamics between the type of question and the type of answer. We have illustrated only the 9 most common combinations (used more than 100 times), including any other answer type or combination in the “other” group. Consistent with the type of question asked by GPs, most answers concerned (differential) diagnosis, which was often combined with responses about referral, further diagnostics, or a combination of these 3 responses. However, the type of question posed by GPs did not always lead to answers within the same topic, such as questions about referral often leading to advice about how to proceed (eg, perform further diagnostics and refer, GP-based follow-up, or start therapy and refer). In this way, one can see that simple referral questions can lead to varied advice possibilities (Multimedia Appendices 6-8).

## Discussion

### Principal Findings

This mixed methods study has shown the growth and evolution of a digital interdisciplinary consultation platform over almost 2 years. Posted questions not only covered a broad spectrum of the population by age and sex but also covered a wide variety of specialist topics. Of note, there was a steep increase in the number of cases for dermatology, which could be explained by existing familiarity with tele-dermatology in Dutch primary care [10] or potentially highlight a practice weakness among GPs.

In most cases, 2 or more users engaged with the GP who initiated the question. An exception to this was the dermatology tile, in which it was typical for only 1 other user to respond. The number of involved specialisms also differed between tiles, being largest for internal medicine. This illustrates a novelty of this approach compared with other consultation formats where a GP only has contact with 1 medical specialist. This approach is in line with the future vision to build primary and secondary care networks around the patient [16,22,23].

The short response times suggest that the Prisma platform facilitates rapid and efficient consultation. This contrasts with telephone consultations, which are often hampered by mutual unavailability. Our data indicate that answers are given to most questions by the end of a GP’s working day so that patient care is not delayed for more than a few hours.

Although it is difficult to compare our study with previous studies because of the difference in design of the platform that was analyzed, the time response outcomes are superior to those in previous studies [4,6,11,24]. It should be considered that they may reflect a precursor effect of enthusiasm among engaged specialists.

The differences in question type between tile categories may indicate differences in work content. Internal medicine, observation, and dermatology focused on diagnosis; surgery focused on treatment; and female/child focused on medication. An alternative hypothesis could be that different specialisms have specific needs of GPs in the treatment process.

The Sankey graph in Figure 5 and Multimedia Appendices 6-8 illustrates the dynamics between questions asked by GPs and answers given by specialists. The large number of questions related to diagnosis had multiple combinations with other questions, reflecting the complexity of evaluation (eg, when the diagnosis is unclear, the next step is also uncertain). Overall, (differential) diagnosis was the most frequently used theme, but this does not appear as a separate group in the graph because it was mostly used in combination with other themes. In comparison to this, questions on medication had most single questions and a clear dynamic to single answers.

The dynamics on referral questions are also interesting, with only a minority of questions receiving a single answer about referral. For example, we found combinations of advice for additional diagnostics in primary care or advice to refer with explanations about diagnosis. We hypothesize that medical specialists used this platform not only to ensure adequate referral but also to share knowledge. There was also a difference between referral questions and answers: not all questions about referral led to answers about referral, and vice versa (ie, referral advice was sometimes given without a specific request).

We found similarities and differences when comparing our findings with the limited amount of preceding research on electronic consultations [15]. In this earlier research, most questions for hematology and rheumatology concerned diagnosis, while questions in the infectious disease and dermatology categories typically concerned therapy. Another research focusing specifically on internal medicine in a hospital in Netherlands involved one-on-one electronic consultations, and revealed “diagnostic tools” to be the most common answer [6].

### Limitations

First, the large sample size and categorization means that a more detailed analysis by specialty is missing in this study. Second, because structure was lacking in the questions posted by GPs, complete data on patient characteristics cannot be guaranteed; however, this did not impair the content analysis. Third, text coding was done by 20 different coders, which might have resulted in interobserver variations in interpretation, despite our efforts to minimize this as much as possible through teamwork. Finally, the data in this analysis were observational in nature, preventing us from making firm conclusions on either observed correlations or patient outcomes.

### Future Research

This evaluation focused on the activities of health care professionals, but to date, the patient perspective has not been analyzed. Although the platform performs well in supporting the needs of the GP for further assessment, treatment, and when needed, more appropriate referral to specialists, we do not know how these relate to needs, experiences, and outcomes in patient cohorts. To generate and implement a novel health care collaboration on a large scale, time and cost-efficiency calculations will also be indispensable [25]. In our study, the response time was more rapid than previously reported for e-consultations [6,24], which have already been shown to reduce not only waiting times for GPs and patients but also costs for patients and waiting lists for hospitals [26]. We are currently conducting a stepped-wedge randomized controlled trial to evaluate the impact of the Prisma platform on patient outcomes and referrals to specialists.

Concerning the content of questions posted on the Prisma platform, an in-depth analysis could still be interesting and useful. Gaps in support for GPs could be uncovered by exploring diagnostic uncertainties (between noncomplex symptoms that meet ICPC diagnostic criteria and practice guidelines), common reasons for referral, and the impact of regional agreements [27]. It is possible that these gaps could be filled by creating a database of information collected on the platform. This could facilitate GPs to ask questions and search for possible answers based on prior responses.

### Conclusion

This observational research shows that a new digital platform facilitated rapid and interactive communication between GPs and specialists for nonurgent questions. This platform is clearly distinguished from one-to-one consultations by facilitating the involvement of multiple physicians. The platform supports the transfer of knowledge from medical specialists to GPs while allowing different viewpoints from relevant experts.

## Appendix

Multimedia Appendix 1 Code tree (in Dutch).

Multimedia Appendix 2 Flowchart inclusion cases.

Multimedia Appendix 3 Characteristics of cases posted in different tiles.

Multimedia Appendix 4 International Classification in Primary Care codes used in cases.

Multimedia Appendix 5 Type of questions and type of answers.

Multimedia Appendix 6 Sankey diagram for differential diagnostic questions. DT: drug treatment; NDT: nondrugs treatment.

Multimedia Appendix 7 Sankey diagram for drug treatment questions.

Multimedia Appendix 8 Sankey diagram for referral questions.

## Abbreviations

GP: general practitioner
ICPC: International Classification in Primary Care

## References

[1] van Oostrom SH, Picavet HSJ, de Bruin SR, Stirbu I, Korevaar JC, Schellevis FG, Baan CA (2014). Multimorbidity of chronic diseases and health care utilization in general practice. BMC Fam Pract.

[2] Schäfer Willemijn L A, Boerma W, Spreeuwenberg P, Schellevis F, Groenewegen P (2016). Two decades of change in European general practice service profiles: conditions associated with the developments in 28 countries between 1993 and 2012. Scand J Prim Health Care.

[3] Kroneman M, Boerma W, van den Berg Michael, Groenewegen P, de Jong Judith, van Ginneken Ewout (2016). Netherlands: Health System Review. Health Syst Transit.

[4] Liddy C, Moroz I, Afkham A, Keely E (2018). Sustainability of a Primary Care-Driven eConsult Service. Ann Fam Med.

[5] Berendsen A, Benneker W, Meyboom-de Jong Betty, Klazinga N, Schuling J (2007). Motives and preferences of general practitioners for new collaboration models with medical specialists: a qualitative study. BMC Health Serv Res.

[6] Muris D, Krekels Mariëlle, Spreeuwenberg Anke, Blom Margje, Bergmans Paul, Cals Jochen W L (2020). General practitioners' use of internal medicine e-consultations. Ned Tijdschr Geneeskd.

[7] Wagner EH, Glasgow RE, Davis C, Bonomi AE, Provost L, McCulloch D, Carver P, Sixta C (2001). Quality Improvement in Chronic Illness Care: A Collaborative Approach. The Joint Commission Journal on Quality Improvement.

[8] de Bever S, Bont J, Scherpbier N (2019). Strengthening general practice by extending specialty training?. Br J Gen Pract.

[9] Greenwood-Lee J, Jewett L, Woodhouse L, Marshall D (2018). A categorisation of problems and solutions to improve patient referrals from primary to specialty care. BMC Health Serv Res.

[10] Tensen E, van der Heijden J P, Jaspers M, Witkamp L (2016). Two Decades of Teledermatology: Current Status and Integration in National Healthcare Systems. Curr Dermatol Rep.

[11] Liddy C, Moroz I, Mihan A, Nawar N, Keely E (2019). A Systematic Review of Asynchronous, Provider-to-Provider, Electronic Consultation Services to Improve Access to Specialty Care Available Worldwide. Telemed J E Health.

[12] Soriano Marcolino Milena, Minelli Figueira Renato, Pereira Afonso Dos Santos Julia, Silva Cardoso Clareci, Luiz Ribeiro Antonio, Alkmim M (2016). The Experience of a Sustainable Large Scale Brazilian Telehealth Network. Telemed J E Health.

[13] Kwok J, Olayiwola J, Knox M, Murphy E, Tuot D (2017). Electronic consultation system demonstrates educational benefit for primary care providers. J Telemed Telecare.

[14] McIntyre T, Kelly E, Clarke T, Green C (2020). Design and implementation of an acute Trauma and Orthopaedic ePlatform (TOP) referral system utilising existing secure technology during the COVID-19 pandemic. Bone & Joint Open.

[15] Waugh M, Voyles D, Thomas M (2015). Telepsychiatry: Benefits and costs in a changing health-care environment. Int Rev Psychiatry.

[16] Miller R, Scherpbier N, van Amsterdam L, Guedes V, Pype P (2019). Inter-professional education and primary care: EFPC position paper. Prim Health Care Res Dev.

[17] Security Whitepaper. Siilo.

[18] Ezra O, Toren A, Tadmor O, Katorza E (2020). Secure Instant Messaging Application in Prenatal Care. J Med Syst.

[19] Friese S (2015). Atlas.ti 8, Windows User Manual. Atlasti.

[20] (2014). International Classification of Primary Care, Second Edition (ICPC-2). ICPC.

[21] Bock NW, Wouters H, Lammers AJ, Blanker MH (2021). Online Consultations Between General Practitioners and Psychiatrists in the Netherlands: A Qualitative Study. Front Psychiatry.

[22] Reddy S (2016). Integrated health care: it’s time for it to blossom. Aust. Health Review.

[23] World Health Organization (WHO) (2015). WHO global strategy on people-centred and integrated health services. WHO.

[24] Ahmed S, Kelly Y, Behera T, Zelen M, Kuye I, Blakey R, Goldstein Sa, Wasfy Jh, Erskine A, Licurse A, Mendu Ml (2020). Utility, Appropriateness, and Content of Electronic Consultations Across Medical Subspecialties. Annals of Internal Medicine.

[25] Liddy C, Drosinis P, Deri Armstrong Catherine, McKellips F, Afkham A, Keely E (2016). What are the cost savings associated with providing access to specialist care through the Champlain BASE eConsult service? A costing evaluation. BMJ Open.

[26] Keely E, Liddy C, Afkham A (2013). Utilization, benefits, and impact of an e-consultation service across diverse specialties and primary care providers. Telemed J E Health.

[27] Dekhuijzen PNR, Smeele IJM, Smorenburg SM, Werkgroep Ketenzorg COPD (2006). [Guideline for the non-pharmacological treatment of COPD]. Ned Tijdschr Geneeskd.

