# Supplementary material for: Evaluation of the First Year(s) of Physicians Collaboration on an Interdisciplinary Electronic Consultation Platform in the Netherlands: Mixed Methods Observational Study
Source: JMIR Hum Factors. 2022 Apr 1;9(2):e33630. doi: 10.2196/33630 (PMC9015779; doi:10.2196/33630)
Supplement: Multimedia Appendix 1 [file humanfactors_v9i2e33630_app1.pdf]

Code	Code definition	Code toelichting
LFT	leeftijd	plaats codes: aan geselecteerde tekst
Man	geslacht man	plaats codes: aan geselecteerde tekst
Vrouw	geslacht vrouw	plaats codes: aan geselecteerde tekst
PT	geslacht onbekend	huisarts benoemt niet of het man of vrouw betreft, niet te achterhalen
Afkomst	Afkomst indien anders dan Nederlands	
VR_kl_ICPC	klacht of diagnose waarover de vraag gesteld wordt	Gebruik de best passende ICPC code, op niveau klacht, of op niveau al gestelde diagnose - zie losse lijst
VG_XXX	voorgeschiedenis	Reeds gestelde diagnoses conform ICPC codering - zie losse lijst. operatieve voorgeschiedenis apart coderen. blanco voorgeschiedenis niet met icpc coderen
OK_XXX	operaties in de voorgeschiedenis	Operaties naar tractus en/of soort aandoening - zie losse lijst
VR_XXX	**SOORT VRAAG**	Een vraag kan meerdere soorten bevatten.
VR_diag	soort vraag diagnostisch	huisarts vraagt bijv. interpretatie van uitslagen, interpretatie van klachten, een dd,
VR_med	soort vraag betreft medicatie	intentie van huisarts was reeds een medicatie beleid en huisarts vraagt specifiek om medicatie advies bijv. start, stop, wijzigen, combinaties, bijwerkingen. dus huisarts vraagt om advies ov
VR_ther	soort vraag therapeutisch	Therapeutische vraag, behandeling starten, anders dan medicatie
VR_ther_med	huisarts vraagt advies of het uberhaupt zinvol is om medicatie te starten als therapie voor de patient	huisarts wil advies over mogelijkheid om medicatie te starten, en zo ja welke
VR_verwijs	soort vraag verwijzing	Vraag over verwijzingen (nodig, richting)
VR_beloop	soort vraag beloop/prognose	Wat is het beloop van de aandoening waarover vraag wordt gesteld
VR_FU	soort vraag over vervolg	hoe moet actief vervolgd worden, welk interval, plaats, persoon
VR_overig	soort vraag overig	Overige vraag, niet te vatten onder diagnostisch, medicatie, therapeutisch of verwijzing
VR_diagnostiek	soort vraag diagnostiek	Huisarts vraagt bijv. of hij diagnostiek moet doen, wat voor diagnostiek
VR_niet_pgb	niet patient gebonden vraag	De vraag gaat niet over een specifieke patient maar is meer algemeen van aard
	VOLLEDIGHEID VRAAG	is de casusschets volledig?
VR_volledig	de casusschets bevat voldoende informatie om de vraag te kunnen beantwoorden	plaats code: titel van de casus
VR_onvolledig	specialist(en) hebben aanvullende essentiele info nodig om vraag te kunnen beantwoorden	plaats code: aan geselecteerde tekst
VR_doorvraag	specialist(en) stellen specifieke aanvullende vragen, waarbij de casus niet direct onvolledig hoeft te zijn.	plaats code: aan geselecteerde tekst
	SOORT ANTWOORD	plaats codes: aan geselecteerde tekst. Deze codes kunnen meerdere keren per casus gebruikt worden
ADV_diagnostiek	advies om diagnostiek uit te voeren	Onder diagnostiek verstaan we bijvoorbeeld ook lab of beeldvorming tijdens follow-up
ADV_medicatie	advies medicamenteuze behandeling	starten, staken, combinatie van medicatie
ADV_therapie_overig	advies overige therapie	overige therapie/behandeling dan medicatie, of andere aanvullende adviezen, tips voor de behandeling
ADV_DD	Meedenken over differentiaaldiagnose	Wanneer de specialist meedenkt over de mogelijke diagnoses, vaak met argumenten voor en tegen verscheidende mogelijke diagnoses
ADV_verwijzing_1eLijn	advies om te verwijzen voor verder beleid in eerstelijn	denk aan verwijzing logopedie, fysiotherapie, psycholoog, thuiszorg inzetten etc.
ADV_verwijzing_2eLijn	advies om te verwijzen voor verder beleid in tweedelijn	verwijzing door huisarts naar tweede lijn of huisarts overlegt met andere specialist buiten prisma
ADV_verwijzing_beh	advies om te overleggen met eigen behandelaar van patient	dit betreft dus niet een nieuwe verwijzing naar de 2e lijn. patient onder behandeling bij deze specialist.
ADV_terug_2e lijn	advies om patient die niet meer onder controle was, terug te verwijzen naar 2e lijn	dit betreft dus een nieuwe verwijzing of overleg, maar patient reeds eerder bekend bij specialist
ADV_follow up	advies om patient terug te zien voor herbeoordeling van de klacht	specialist adviseert aan huisarts patient te volgen, even aan te kijken
ADV_verdieping_ha	advies om huisarts meer vragen te laten stellen aan patient voor verduidelijking van klacht	specialist adviseert aan huisarts om klachten verder uit te diepen nav overwegingen van specialisten (welke dd)
ADV_expectatief	advies om af te wachten	specialist adviseert huisarts om af te wachten
ADV_overig	soort advies overig	wanneer het niet te vatten is onder diagnostisch, medicatie etc.
ADV_afraden	advies om expliciteit geen behandeling, diagnostiek of verwijzing te doen	
UITLEG_diagnose	antwoord bevat duiding van de diagnose obv de geleverde informatie	
UITLEG_verdieping	antwoord bevat verdere verdieping of uitleg van achtergronden	meestal in aanvulling op uitleg diagnose, nog nadere informatie over achtergronden, beloop
ANTWOORD_PRISMA	huisarts heeft soortgelijke casus gevonden	bijv via zoekfunctie of andere chat waardoor huisarts verder kan met casus
ANTWOORD_ZELF	casus is inmiddels zelf opgelost door huisarts /patient	zonder gebruik te maken van prisma
ANTWOORD_GEEN	er komt geen enkele reactie van specialisten	
	OVEREENKOMSTEN / VERSCHILLEN TUSSEN SPECIALISTEN	plaats codes: aan geselecteerde tekst
ADV_eens	antwoord komt overeen met eerder antwoord binnen casus	
ADV_oneens	antwoord is duidelijk niet in lijn met eerder antwoord binnen casus	
ADV_deels_eens	antwoord gedeeltelijk eens, gedeeltelijk oneens met eerder antwoord	specialist is slecht met een deel van antwoord collega eens, maar duidelijk oneens met overig deel
ADV_toevoeging	nadrukkelijke toevoeging op eerder advies	specialist is eens met antwoord van collega en vult nog extra aan met niet eerdere gegeven advies
ADV_herhaling	De specialist is het eens met zijn collega, maar herhaalt het advies in zijn eigen woorden	Bij herhaling is het advies inhoudelijk hetzelfde, maar net wat uitgebreider of juist bondiger en in net andere woorden
	VERVOLG OP ANTWOORDEN	plaats codes: aan geselecteerde tekst. Deze codes kunnen meerdere keren per casus gebruikt worden
NWE_vraag	het antwoord van de specialist levert een nieuwe vraag op bij de huisarts	Deze nieuwe vraag hoeft niet gescoord te worden op soort etc
DISCUSSIE	de casus / het antwoord leidt tot discussie tussen gebruikers	dit kan eens of oneens zijn, of een nieuwe discussie of verdieping
NWE_HA	tweede huisarts antwoordt/denkt mee	een andere huisarts 'mengt zich' in het gesprek.
VR_nwedeelnemer	Een specialist of huisarts stelt een (algemene) vraag aan de hand van de casus	Het antwoord resulteert vaak in een leereffect
	VERDIEPING	plaats codes: aan geselecteerde tekst
LEEREFFECT_HA		tijdens discussie wordt genoemd dat er iets nieuws is geleerd door een van de deelnemers (huisarts)
LEEREFFECT_SP		tijdens discussie wordt genoemd dat er iets nieuws is geleerd door een van de deelnemers (specialist), of blijk van 'INTERESSE'
ACHTERGROND	specialist maakt in tekst een link of verwijzing naar een relevant artikel	voor achtergrond informatie, informatieve webstie of stroomdiagram, maar geen richtlijn
RICHTLIJN_HA	specialist of huisarts maakt in tekst een link of verwijzing naar de huisartsenrichtlijn (NHG)	
RICHTLIJN_SP	specialist of huisarts maakt in tekst een link of verwijzing naar de specialistenrichtlijn	dit kan nederlands of internationaal zijn
	COMPLEXITEIT	plaats code: titel van de casus
complex_JA	[indien veel vragen werden gesteld om meer informatie te verkrijgen en/of de antwoorden van de specialisten verschilden sterk en leidde dit tot discussie onderling]	
complex_NEE	[indien het een eenvoudige vraag was waarop een eenvoudig antwoord werd gegeven]	er kan een uitgebreid verhaal omheen zijn (voorgeschiedenis), maar vraag en antwoord zijn kort/eenvoudig
complex_TWIJFEL	Niet duidelijk bij JA of NEE in te delen	NB probeer een toelichting te geven waarom je twijfelt
	**BRUIKBAARHEID**	**plaats codes: aan geselecteerde tekst**
bruikbaarheid_JA	huisarts benoemt dat hij/zij met de casus verder kan of is geholpen met de adviezen	DANK vanuit de huisarts volstaat ook als bruikbaar
bruikbaarheid_NEE	huisarts benoemt dat hij/zij niet verder kan of niet is geholpen	
bruikbaarheid_TWIJFEL	huisarts benoemt niets of er volgt geen antwoord/afsluiting door de huisarts	bij bruikbaarheid twijfel kan onderzoeker toevoegen of vraag wel of niet is beantwoord
beantwoord_JA	naar oordeel van onderzoeker is de concrete vraag van de huisarts in de adviezen beantwoord	indien huisarts niets terugkoppelt, kan deze code worden gebruikt
beantwoord_NEE	naar oordeel van onderzoeker is de concrete vraag van de huisarts in de adviezen niet beantwoord	indien huisarts niets terugkoppelt, kan deze code worden gebruikt
afsluiting_JA	huisarts benoemt dat hij/zij de casus volledig heeft afgerond	patient hoeft, voor deze vraag, niet terug te komen bij de huisarts
afsluiting_NEE	huisarts benoemt dat hij/zij de casus nog gaat volgen, uitdiepen, follow up	
terugkoppeling_vraag	specialisten verzoeken terugkoppeling van de casus na vervolg advies	
terugkoppeling_ant	huisarts koppelt terug na vervolg advies	
	PATIENTEN OORDEEL	plaats codes: aan geselecteerde tekst
patientinfo_ja	huisarts meldt dat hij/zij patient heeft verteld dat de casus wordt voorgelegd aan team specialisten	
patientinfo_onbekend	huisarts meldt niets mbt informeren van patient over gebruik van prisma	
patient_tevreden	huisarts benoemt tevredenheid van patient over advies van specialisten	
patient_ontevreden	huisarts benoemt ontevredenheid van patient over advies van specialisten	hierbij ook vervolg stappen van huisarts coderen (bijv alsnog doorverwijzing 2e lijn)
patient_gerust	huisartsn benoemt dat patient is gerustgesteld over advies van specialisten	
patient_ongerust	huisarts benoemt dat patient niet is gerustgesteld over advies van specialisten	hierbij ook vervolg stappen van huisarts coderen (bijv alsnog doorverwijzing 2e lijn)
	SPECIALISTEN OORDEEL	meerdere codes mogelijk
enthousiast	specialist benoemt enthousiasme over casus/discussie	onderzoeker duidt werkplezier van specialist (bijv : wat een leuke casus, hier wordt ik blij of enthousiast van'.
makkelijk	specialist benoemt dat casus simpel is op te lossen	
ingewikkeld	specialist benoemt dat casus ingewikkeld is	
	OVERIG	
BIJLAGE_foto	een foto als bijlage toegevoegd	foto van patient door huisarts bijgevoegd
BIJLAGE_overig	overige bijlagen (geen foto)	bijlage door huisarts zoals lab, brief, beeldvorming
VERKEERD	verkeerde tegel, casus had in andere tegel gemoeten	

ver de medicatie

Code	Code definition	Code group
VG_ICPC	klacht of diagnose waarover de vraag gesteld wordt	
VG_A70	Gegeneraliseerde tuberculose [ex. R70]	
VG_A71	Mazelen	
VG_A72	Waterpokken	
VG_A73	Malaria	
VG_A74	Rode hond	
VG_A75	Mononucleosis infectiosa	
VG_A76	Andere virusziekte met exantheem	
VG_A77	Andere virusziekte(n)	
VG_A78	Andere infectieziekte(n)	
VG_A79	Maligniteit met onbekende primaire lokalisatie	
VG_A80	Trauma/letsel	
VG_A81	Multiple traumata/inwendig letsels	
VG_A82	Laat gevolg van letsel	
VG_A84	Geneesmiddelintoxicatie	
VG_A85	Geneesmiddelbijwerking	
VG_A86	Intoxicatie andere chemische stof	
VG_A87	Complicatie(s) medische behandeling	
VG_A88	Schadelijk gevolg fysische factor [ex. H85]	
VG_A89	Aanwezigheid/gevolg prothese	
VG_A90	Multiple aangeboren afwijkingen	
VG_A91	Afwijkende uitslag(en) onderzoek	
VG_A92	Toxoplasmose	
VG_A93	Dysmatuur/prematuur/immatuur levendgeborene	
VG_A94	Perinatale morbiditeit	
VG_A95	Perinatale mortaliteit	
VG_A96	Dood/overlijden [ex. A95]	
VG_A97	Geen ziekte	
VG_A99	Andere gegeneraliseerde/niet gespecificeerde ziekte(n)	
VG_B70	Acute lymphadenitis	
VG_B71	Chronische/niet-gespecificeerde lymphadenitis	
VG_B72	Ziekte van Hodgkin	
VG_B73	Leukemie	
VG_B74	Andere maligniteit bloed/lymfestelsel	
VG_B75	Benigne/niet-gespecificeerd neoplasma bloed/lymfestelsel	
VG_B76	Miltruptuur	
VG_B77	Ander letsel bloed/lymfestelsel	
VG_B78	Erfelijke hemolytische anemie	
VG_B79	Andere aangeboren afwijking bloed/lymfestel	
VG_B80	IJzergebrekanemie	
VG_B81	Pernicieuze/foliumzuurdeficiëntie-anemie	
VG_B82	Andere/niet-gespecificeerde anemie	
VG_B83	Purpura/stollingsstoornis/afwijkende trombocyten	
VG_B84	Afwijking leukocyten	
VG_B85	Onverklaarde afwijking bloedonderzoek	
VG_B86	Andere hematologische afwijking(en)	
VG_B87	Splenomegalie	
VG_B90	HIV-infectie (AIDS/ARC)	
VG_B99	Andere ziekte(n) bloed/lymfestelsel/milt	
VG_D70	Infectieuze diarree, dysenterie	
VG_D71	Bof	
VG_D72	Virus hepatitis	
VG_D73	Veronderstelde gastro-intestinale infectie	
VG_D74	Maligniteit maag	
VG_D75	Maligniteit colon/rectum	
VG_D76	Maligniteit pancreas	
VG_D77	Andere/niet-gespecif.maligniteit spijsverteringsorganen	
VG_D78	Benigne neoplasma spijsverteringsorganen	
VG_D79	Corpus alienum via mond/anus	
VG_D80	Ander letsel spijsverteringsorganen	
VG_D81	Aangeboren afwijking(en) spijsverteringsorganen	
VG_D82	Ziekte tanden/tandvlees	
VG_D83	Ziekte mond/tong/lippen/speekselklieren	
VG_D84	Ziekte oesofagus	
VG_D85	Ulcus duodeni	
VG_D86	Ander ulcus pepticum	
VG_D87	Stoornis maagfunctie	
VG_D88	Appendicitis	
VG_D89	Hernia inguinalis	
VG_D90	Hernia diafragmatica/hiatus	
VG_D91	Andere hernia abdominalis	
VG_D92	Diverticulose/diverticulitis	
VG_D93	Prikkelbare darm syndroom	
VG_D94	Colitis ulcerosa/chronische enteritis (regionalis)	
VG_D95	Fissura ani/perianaal abces	
VG_D96	Hepatomegalie	
VG_D97	Cirrose/andere leverziekte	
VG_D98	Cholecystitis/cholelithiasis	
VG_D99	Andere ziekte(n) spijsverteringsorganen	
VG_F70	Infectieuze conjunctivitis	
VG_F72	Blepharitis/hordeolum/chalazion	
VG_F73	Andere infectie/ontsteking oog/adnexen [ex. F85,F86]	
VG_F74	Neoplasma oog/adnexen	
VG_F75	Kneuzing/bloeding oog/adnexen	
VG_F76	Corpus alienum oog	
VG_F79	Ander letsel oog/adnexen	
VG_F80	Stenose traankanaal zuigeling	
VG_F81	Andere aangeboren afwijking(en) oog/adnexen	
VG_F82	Netvliesloslating	
VG_F83	Retinopathie	
VG_F84	Maculadegeneratie	
VG_F85	Ulcus corneae	
VG_F86	Trachoom	
VG_F91	Refractie afwijking(en)	
VG_F92	Cataract/staar	
VG_F93	Glaucoom/verhoogde oogdruk	
VG_F94	Blindheid (elke graad/vorm)	
VG_F95	Strabismus/scheelzien	
VG_F99	Andere ziekte(n) oog/adnexen	

VG_H70	Otitis externa
VG_H71	Otitis media acuta/myringitis
VG_H72	Otitis media met effusie
VG_H73	Tubair catarre/tubastenose
VG_H74	Chronische otitis media/andere infectie oor
VG_H75	Neoplasma oor
VG_H76	Corpus alienum in oor
VG_H77	Perforatie trommelvlies [ex. H71]
VG_H78	Oppervlakkig letsel oor
VG_H79	Ander letsel oor
VG_H80	Aangeboren afwijking(en) oor
VG_H81	Overmatig cerumen
VG_H82	Vertigosyndroom/labyrinthitis [ex. N17]
VG_H83	Otosclerose
VG_H84	Presbyacusis
VG_H85	Akoestisch letsel/lawaaidoofheid
VG_H86	Doofheid/slechthorendheid
VG_H99	Andere ziekte(n) oor
VG_K70	Infectieziekte hartvaatstelsel
VG_K71	Acuut reuma/reumatische hartziekte
VG_K72	Neoplasma hartvaatstelsel
VG_K73	Aangeboren afwijking(en) hartvaatstelsel
VG_K74	Angina pectoris
VG_K75	Acuut myocardinfarct
VG_K76	Andere/chronische ischemische hartziekte
VG_K77	Decompensatio cordis
VG_K78	Boezemfibrilleren/-fladderen
VG_K79	Paroxysmale tachycardie
VG_K80	Ectopische slagen/extrasystolen
VG_K81	Hartgeruis
VG_K82	Cor pulmonale
VG_K83	Niet-reumatische klepaandoening
VG_K84	Andere hartziekte(n)
VG_K85	Verhoogde bloeddruk [ex. K86,K87]
VG_K86	Essentiële hypertensie zonder orgaanbeschadiging
VG_K87	Hypertensie met orgaanbeschadiging/secundaire hypertensie
VG_K88	Orthostatische hypotensie
VG_K89	Passagère cerebrale ischemie/TIA
VG_K90	Cerebrovasculair accident (CVA)
VG_K91	Atherosclerose [ex. K76,K90]
VG_K92	Andere ziekte(n) perifere arteriën
VG_K93	Longembolie/longinfarct
VG_K94	Thrombophlebitis/flebotrombose
VG_K95	Varices benen [ex. S97]
VG_K96	Hemorroïden
VG_K99	Andere ziekte(n) hartvaatstelsel
VG_L70	Infectieziekte bewegingsapparaat
VG_L71	Neoplasma bewegingsapparaat
VG_L72	Fractuur radius/ulna
VG_L73	Fractuur tibia/fibula
VG_L74	Fractuur hand/voet
VG_L75	Fractuur femur
VG_L76	Andere fractuur
VG_L77	Verstuiking/distorsie enkel
VG_L78	Verstuiking/distorsie knie
VG_L79	Andere verstuiking/distorsie
VG_L80	Luxatie/subluxatie
VG_L81	Ander letsel bewegingsapparaat
VG_L82	Aangeboren afwijking(en) bewegingsapparaat
VG_L83	Syndroom cervicale wervelkolom
VG_L84	Artrose/spondylose wervelkolom
VG_L85	Verworven afwijking(en) wervelkolom
VG_L86	Lage-rugpijn met uitstraling
VG_L87	Ganglion gewricht/pees
VG_L88	Reumatoïde artritis/verwante aandoening(en)
VG_L89	Coxartrose
VG_L90	Gonartrose
VG_L91	Andere artrose/verwante aandoening(en)
VG_L92	Schoudersyndroom/PHS
VG_L93	Epicondylitis lateralis
VG_L94	Osgood-Schlatter/andere osteochondropathie
VG_L95	Osteoporose
VG_L96	Acuut letsel meniscus/kniebanden
VG_L97	Chronisch inwendig trauma knie
VG_L98	Verworven afwijking(en) extremiteiten
VG_L99	Andere ziekte(n) bewegingsapparaat
VG_N70	Poliomyelitis/andere enterovirus infectie
VG_N71	Meningitis/encephalitis
VG_N72	Tetanus
VG_N73	Andere infectieziekte(n) zenuwstelsel
VG_N74	Maligniteit zenuwstelsel
VG_N75	Benigne neoplasma zenuwstelsel
VG_N76	Niet-gespecificeerd neoplasma zenuwstelsel
VG_N79	Hersenschudding/hoofdtrauma
VG_N80	Ander letsel hoofd [ex. L76]
VG_N81	Ander letsel zenuwstelsel
VG_N85	Aangeboren afwijking(en) zenuwstelsel
VG_N86	Multiple sclerose
VG_N87	Parkinsonisme, ziekte van Parkinson
VG_N88	Epilepsie (alle vormen)
VG_N89	Migraine
VG_N90	Cluster headache
VG_N91	Facialis parese/Bell's palsy
VG_N92	Trigeminus neuralgie
VG_N93	Carpale tunnelsyndroom
VG_N94	Andere perifere neuritis/neuropathie
VG_N99	Andere ziekte(n) zenuwstelsel
VG_P70	Seniele dementie/Alzheimer
VG_P71	Andere organische psychose(n)
VG_P72	Schizofrenie

VG_P73	Affectieve psychose
VG_P74	Angststoornis/angsttoestand
VG_P75	Somatisatiestoornis
VG_P76	Depressie
VG_P77	Suïcidepoging
VG_P78	Neurasthenie/surmenage
VG_P79	Andere neurose
VG_P80	Persoonlijkheids-/karakterstoornis
VG_P85	Mentale retardatie/intellectuele achterstand
VG_P98	Andere/niet gespecificeerde psychose(n)
VG_P99	Andere psychische stoornissen
VG_R70	Tuberculose luchtwegen [ex. A70]
VG_R71	Kinkhoest
VG_R72	Streptokokken-angina/roodvonk
VG_R73	Furunkel/abces neus
VG_R74	Acute infectie bovenste luchtwegen
VG_R75	Acute/chronische rinosinusitis
VG_R76	Acute tonsillitis/peritonsillair abces
VG_R77	Acute laryngitis/tracheïtis
VG_R78	Acute bronchitis/bronchiolitis
VG_R80	Influenza [ex. R81]
VG_R81	Pneumonie
VG_R82	Pleuritis alle vormen [ex. R70]
VG_R83	Andere infectie(s) luchtwegen
VG_R84	Maligniteit bronchus/long
VG_R85	Andere maligniteit luchtwegen
VG_R86	Benigne neoplasma luchtwegen
VG_R87	Corpus alienum neus/larynx/bronchiën
VG_R88	Ander letsel luchtwegen
VG_R89	Aangeboren afwijking(en) luchtwegen
VG_R90	Hypertrofie/chronische infectie tonsillen/adenoïd
VG_R91	Chronische bronchitis/bronchiëctasieën
VG_R93	Pleuravocht
VG_R95	Emfyseem/COPD
VG_R96	Astma
VG_R97	Hooikoorts/allergische rinitis
VG_R98	Hyperventilatie
VG_R99	Andere ziekte(n) luchtwegen
VG_S70	Herpes zoster
VG_S71	Herpes simplex [ex. F85,X90,Y72]
VG_S72	Scabies/andere aandoening door mijten
VG_S73	Pediculosis/andere huidinfestatie
VG_S74	Dermatomycose(n)
VG_S75	Moniliasis/candidiasis [ex. X72,Y75]
VG_S76	Andere infectie huid/subcutis
VG_S77	Maligniteit huid/subcutis
VG_S78	Lipoom
VG_S79	Ander benigne neoplasma huid/subcutis
VG_S80	Ander/niet-gespecificeerd neoplasma huid/subcutis
VG_S81	Hemangioom/lymfangioom
VG_S82	Naevus/moedervlek
VG_S83	Andere aangeboren afwijking(en) huid/subcutis
VG_S84	Impetigo/impetiginisatie
VG_S85	Pilonidaal cyste/fistel
VG_S86	Seborroïsch eczeem/roos
VG_S87	Constitutioneel eczeem
VG_S88	Contact eczeem/ander eczeem
VG_S89	Luiereczeem
VG_S90	Pityriasis rosea
VG_S91	Psoriasis
VG_S92	Ziekte(n) zweetklieren
VG_S93	Atheroomcyste/epitheelcyste
VG_S94	Unguis incarnatus/andere nagelaandoening
VG_S95	Mollusca contagiosa
VG_S96	Acne
VG_S97	Ulcus cruris/decubitus/chronisch ulcus
VG_S98	Urticaria
VG_S99	Andere ziekte(n) huid/subcutis
VG_T70	Infectie endocriene klier(en)
VG_T71	Maligniteit schildklier
VG_T72	Benigne neoplasma schildklier
VG_T73	Ander/niet-gespecificeerd neoplasma endocriene klieren
VG_T78	Persisterende ductus thyreoglossus/cyste
VG_T80	Andere aangeboren afwijking endocriene klieren/metabolisme
VG_T81	Struma/noduli [ex. T85,T86]
VG_T82	Adipositas
VG_T83	Overgewicht
VG_T85	Hyperthyreoïdie/thyreotoxicose
VG_T86	Hypothyreoïdie/myxoedeem
VG_T87	Hypoglykemie
VG_T88	Renale glucosurie
VG_T90	Diabetes mellitus
VG_T91	Vitamine-/voedingsdeficiëntie(s)
VG_T92	Jicht
VG_T93	Vetstofwisselingsstoornis(sen)
VG_T99	Andere ziekte(n) endocriene klieren/metabolisme/voeding
VG_U70	Acute pyelonephritis/pyelitis
VG_U71	Cystitis/urineweginfectie
VG_U72	Niet specifieke urethritis [ex. X99,Y99]
VG_U75	Maligniteit nier
VG_U76	Maligniteit blaas
VG_U77	Andere maligniteit urinewegen
VG_U78	Benigne neoplasma urinewegen
VG_U79	Niet-gespecificeerd neoplasma urinewegen
VG_U80	Letsel urinewegen
VG_U85	Aangeboren afwijking(en) urinewegen
VG_U88	Glomerulonephritis/nefrose
VG_U90	Orthostatische proteïnurie
VG_U95	Urolithiasis (alle vormen/lokalisaties)
VG_U98	Afwijkende uitslag urine-onderzoek

VG_U99	Andere ziekte(n) urinewegen
VG_W70	Puerperale infectie/sepsis
VG_W71	Andere infectie(s) zwangerschap/kraambed [ex. W70]
VG_W72	Maligniteit in verband met zwangerschap
VG_W73	Benigne neoplasma in verband met zwangerschap
VG_W75	Zwangerschap complicerende letsels
VG_W76	Zwangerschap complicerende aangeboren afwijking moeder
VG_W77	Zwangerschap complicerende niet-obstetrische factor
VG_W78	Zwangerschap: bevestigd
VG_W79	Ongewenste zwangerschap: bevestigd
VG_W80	Ectopische zwangerschap
VG_W81	Toxicose/(pre-)eclampsie
VG_W82	Spontane abortus
VG_W83	Abortus provocatus
VG_W84	Zwangerschap met verhoogd risico
VG_W90	Normale bevalling levendgeborene
VG_W91	Normale bevalling doodgeborene
VG_W92	Gecompliceerde bevalling levendgeborene
VG_W93	Gecompliceerde bevalling doodgeborene
VG_W94	Mastitis puerperalis
VG_W95	Andere aandoening borsten kraambed
VG_W96	Andere complicatie(s) kraambed
VG_W99	And. ziekte(n) ivm zwangersch./beval./kraamb./anticonceptie
VG_X70	Lues vrouw [ex. A90]
VG_X71	Gonorroe vrouw
VG_X72	Candidiasis urogenitale vrouw
VG_X73	Trichomonas urogenitale vrouw
VG_X74	Ontsteking kleine bekken/PID
VG_X75	Maligniteit cervix uteri
VG_X76	Maligniteit borst vrouw
VG_X77	Andere maligniteit geslachtsorganen vrouw
VG_X78	Benigne neoplasma uterus/cervix uteri
VG_X79	Benigne neoplasma borsten vrouw [ex. X88]
VG_X80	Ander benigne neoplasma geslachtsorganen vrouw
VG_X81	Ander/niet-gespecificeerd neoplasma geslachtsorganen vrouw
VG_X82	Letsel geslachtsorganen vrouw
VG_X83	Aangeboren afwijking(en) geslachtsorganen vrouw
VG_X84	Vaginitis/vulvitis nao
VG_X85	Cervicitis/andere ziekte cervix
VG_X86	Afwijkende cervixuitstrijk
VG_X87	Prolaps vagina/uterus
VG_X88	Fibroadenoom/polycystische afwijking borsten
VG_X89	Premenstrueel spanningssyndroom
VG_X90	Herpes genitalis vrouw
VG_X91	Condylomata acuminata vrouw
VG_X99	Andere ziekte(n) geslachtsorganen/borsten vrouw
VG_Y70	Lues man [ex. A90]
VG_Y71	Gonorroe man
VG_Y72	Herpes genitalis man
VG_Y73	Prostatitis/vesiculitis seminalis
VG_Y74	Orchitis/epididymitis
VG_Y75	Balanitis
VG_Y76	Condylomata acuminata man
VG_Y77	Maligniteit prostaat
VG_Y78	Andere maligniteit geslachtsorganen/borsten man
VG_Y79	Benigne neoplasma geslachtsorganen/borsten man
VG_Y80	Letsel geslachtsorganen man
VG_Y81	Phimosis/slurf-preputium
VG_Y82	Hypospadie
VG_Y83	Cryptorchisme/niet ingedaalde testis
VG_Y84	Andere aangeboren afwijking(en) geslachtsorganen/borsten man
VG_Y85	Benigne prostaathypertrofie
VG_Y86	Hydrokèle
VG_Y99	Andere ziekte(n) geslachtsorganen/borsten man

	OPERATIES IN VOORGESCHIEDENIS
OK_KNO	operatie KNO gebied
OK_cardiovasculair	operatie aan hart of bloedvaten
OK_orthopedie	operatie aan botten
OK_tractus digestivus	operatie aan maagdarmstelsel, pancreas, lever, galwegen
OK_gynaecologisch	operatie aan uterus, ovariae
OK_urologisch	operatie aan blaas, nieren, prostaat
OK_neurochirurgie	operatie aan hersenen, zenuwen
OK_oncologie	operatie van tumoren
OK_mamma	operatie van mamma(e)
OK_oogheelkunde	operatie van de ogen
OK_dermatologie	operatie van de huid
VG_radiotherapie	radiotherapie
VG_chemotherapie	chemotherapie

toelichting
Reeds gestelde diagnoses (voorgeschiedenis / comorbiditeit)

operaties, indien vermeld, in de voorgeschiedenis worden gelabeld op soort aandoening en tractus

ook sectio cesaria

tevens orgaangebied coderen

Code
VR_kl_ICPC
VR_kl_A01
VR_kl_A02
VR_kl_A03
VR_kl_A04
VR_kl_A05
VR_kl_A06
VR_kl_A07
VR_kl_A08
VR_kl_A09
VR_kl_A10
VR_kl_A12
VR_kl_A13
VR_kl_A14
VR_kl_A15
VR_kl_A16
VR_kl_A17
VR_kl_A20
VR_kl_A25
VR_kl_A26
VR_kl_A27
VR_kl_A28
VR_kl_A29
VR_kl_A69
VR_kl_A70
VR_kl_A71
VR_kl_A72
VR_kl_A73
VR_kl_A74
VR_kl_A75
VR_kl_A76
VR_kl_A77
VR_kl_A78
VR_kl_A79
VR_kl_A80
VR_kl_A81
VR_kl_A82
VR_kl_A84
VR_kl_A85
VR_kl_A86
VR_kl_A87
VR_kl_A88
VR_kl_A89
VR_kl_A90
VR_kl_A91
VR_kl_A92
VR_kl_A93
VR_kl_A94
VR_kl_A95
VR_kl_A96
VR_kl_A97

VR_kl_A99
VR_kl_B02
VR_kl_B03
VR_kl_B04
VR_kl_B25
VR_kl_B26
VR_kl_B27
VR_kl_B28
VR_kl_B29
VR_kl_B70
VR_kl_B71
VR_kl_B72
VR_kl_B73
VR_kl_B74
VR_kl_B75
VR_kl_B76
VR_kl_B77
VR_kl_B78
VR_kl_B79
VR_kl_B80
VR_kl_B81
VR_kl_B82
VR_kl_B83
VR_kl_B84
VR_kl_B85
VR_kl_B86
VR_kl_B87
VR_kl_B90
VR_kl_B99
VR_kl_D01
VR_kl_D02
VR_kl_D03
VR_kl_D04
VR_kl_D05
VR_kl_D06
VR_kl_D08
VR_kl_D09
VR_kl_D10
VR_kl_D11
VR_kl_D12
VR_kl_D13
VR_kl_D14
VR_kl_D15
VR_kl_D16
VR_kl_D17
VR_kl_D18
VR_kl_D19
VR_kl_D20
VR_kl_D21
VR_kl_D22
VR_kl_D24
VR_kl_D25

VR_kl_D26
VR_kl_D27
VR_kl_D28
VR_kl_D29
VR_kl_D70
VR_kl_D71
VR_kl_D72
VR_kl_D73
VR_kl_D74
VR_kl_D75
VR_kl_D76
VR_kl_D77
VR_kl_D78
VR_kl_D79
VR_kl_D80
VR_kl_D81
VR_kl_D82
VR_kl_D83
VR_kl_D84
VR_kl_D85
VR_kl_D86
VR_kl_D87
VR_kl_D88
VR_kl_D89
VR_kl_D90
VR_kl_D91
VR_kl_D92
VR_kl_D93
VR_kl_D94
VR_kl_D95
VR_kl_D96
VR_kl_D97
VR_kl_D98
VR_kl_D99
VR_kl_F01
VR_kl_F02
VR_kl_F03
VR_kl_F04
VR_kl_F05
VR_kl_F13
VR_kl_F14
VR_kl_F15
VR_kl_F16
VR_kl_F17
VR_kl_F18
VR_kl_F27
VR_kl_F28
VR_kl_F29
VR_kl_F70
VR_kl_F72
VR_kl_F73
VR_kl_F74

VR_kl_F75
VR_kl_F76
VR_kl_F79
VR_kl_F80
VR_kl_F81
VR_kl_F82
VR_kl_F83
VR_kl_F84
VR_kl_F85
VR_kl_F86
VR_kl_F91
VR_kl_F92
VR_kl_F93
VR_kl_F94
VR_kl_F95
VR_kl_F99
VR_kl_H01
VR_kl_H02
VR_kl_H03
VR_kl_H04
VR_kl_H05
VR_kl_H13
VR_kl_H15
VR_kl_H27
VR_kl_H28
VR_kl_H29
VR_kl_H70
VR_kl_H71
VR_kl_H72
VR_kl_H73
VR_kl_H74
VR_kl_H75
VR_kl_H76
VR_kl_H77
VR_kl_H78
VR_kl_H79
VR_kl_H80
VR_kl_H81
VR_kl_H82
VR_kl_H83
VR_kl_H84
VR_kl_H85
VR_kl_H86
VR_kl_H99
VR_kl_K01
VR_kl_K02
VR_kl_K03
VR_kl_K04
VR_kl_K05
VR_kl_K06
VR_kl_K07
VR_kl_K24

VR_kl_K25
VR_kl_K27
VR_kl_K28
VR_kl_K29
VR_kl_K70
VR_kl_K71
VR_kl_K72
VR_kl_K73
VR_kl_K74
VR_kl_K75
VR_kl_K76
VR_kl_K77
VR_kl_K78
VR_kl_K79
VR_kl_K80
VR_kl_K81
VR_kl_K82
VR_kl_K83
VR_kl_K84
VR_kl_K85
VR_kl_K86
VR_kl_K87
VR_kl_K88
VR_kl_K89
VR_kl_K90
VR_kl_K91
VR_kl_K92
VR_kl_K93
VR_kl_K94
VR_kl_K95
VR_kl_K96
VR_kl_K99
VR_kl_L01
VR_kl_L02
VR_kl_L03
VR_kl_L04
VR_kl_L05
VR_kl_L06
VR_kl_L07
VR_kl_L08
VR_kl_L09
VR_kl_L10
VR_kl_L11
VR_kl_L12
VR_kl_L13
VR_kl_L14
VR_kl_L15
VR_kl_L16
VR_kl_L17
VR_kl_L18
VR_kl_L19
VR_kl_L20

VR_kl_L26
VR_kl_L27
VR_kl_L28
VR_kl_L29
VR_kl_L70
VR_kl_L71
VR_kl_L72
VR_kl_L73
VR_kl_L74
VR_kl_L75
VR_kl_L76
VR_kl_L77
VR_kl_L78
VR_kl_L79
VR_kl_L80
VR_kl_L81
VR_kl_L82
VR_kl_L83
VR_kl_L84
VR_kl_L85
VR_kl_L86
VR_kl_L87
VR_kl_L88
VR_kl_L89
VR_kl_L90
VR_kl_L91
VR_kl_L92
VR_kl_L93
VR_kl_L94
VR_kl_L95
VR_kl_L96
VR_kl_L97
VR_kl_L98
VR_kl_L99
VR_kl_N01
VR_kl_N02
VR_kl_N03
VR_kl_N04
VR_kl_N05
VR_kl_N06
VR_kl_N07
VR_kl_N16
VR_kl_N17
VR_kl_N18
VR_kl_N19
VR_kl_N26
VR_kl_N27
VR_kl_N28
VR_kl_N29
VR_kl_N70
VR_kl_N71
VR_kl_N72

VR_kl_N73
VR_kl_N74
VR_kl_N75
VR_kl_N76
VR_kl_N79
VR_kl_N80
VR_kl_N81
VR_kl_N85
VR_kl_N86
VR_kl_N87
VR_kl_N88
VR_kl_N89
VR_kl_N90
VR_kl_N91
VR_kl_N92
VR_kl_N93
VR_kl_N94
VR_kl_N99
VR_kl_P01
VR_kl_P02
VR_kl_P03
VR_kl_P04
VR_kl_P05
VR_kl_P06
VR_kl_P07
VR_kl_P08
VR_kl_P09
VR_kl_P10
VR_kl_P11
VR_kl_P12
VR_kl_P13
VR_kl_P15
VR_kl_P16
VR_kl_P17
VR_kl_P18
VR_kl_P19
VR_kl_P20
VR_kl_P21
VR_kl_P22
VR_kl_P23
VR_kl_P24
VR_kl_P25
VR_kl_P27
VR_kl_P28
VR_kl_P29
VR_kl_P70
VR_kl_P71
VR_kl_P72
VR_kl_P73
VR_kl_P74
VR_kl_P75
VR_kl_P76

VR_kl_P77
VR_kl_P78
VR_kl_P79
VR_kl_P80
VR_kl_P85
VR_kl_P98
VR_kl_P99
VR_kl_R01
VR_kl_R02
VR_kl_R03
VR_kl_R04
VR_kl_R05
VR_kl_R06
VR_kl_R07
VR_kl_R08
VR_kl_R09
VR_kl_R21
VR_kl_R22
VR_kl_R23
VR_kl_R24
VR_kl_R25
VR_kl_R26
VR_kl_R27
VR_kl_R28
VR_kl_R29
VR_kl_R70
VR_kl_R71
VR_kl_R72
VR_kl_R73
VR_kl_R74
VR_kl_R75
VR_kl_R76
VR_kl_R77
VR_kl_R78
VR_kl_R80
VR_kl_R81
VR_kl_R82
VR_kl_R83
VR_kl_R84
VR_kl_R85
VR_kl_R86
VR_kl_R87
VR_kl_R88
VR_kl_R89
VR_kl_R90
VR_kl_R91
VR_kl_R93
VR_kl_R95
VR_kl_R96
VR_kl_R97
VR_kl_R98
VR_kl_R99

VR_kl_S01
VR_kl_S02
VR_kl_S03
VR_kl_S04
VR_kl_S05
VR_kl_S06
VR_kl_S07
VR_kl_S08
VR_kl_S09
VR_kl_S10
VR_kl_S11
VR_kl_S12
VR_kl_S13
VR_kl_S14
VR_kl_S15
VR_kl_S16
VR_kl_S17
VR_kl_S18
VR_kl_S19
VR_kl_S20
VR_kl_S21
VR_kl_S22
VR_kl_S23
VR_kl_S24
VR_kl_S26
VR_kl_S27
VR_kl_S28
VR_kl_S29
VR_kl_S70
VR_kl_S71
VR_kl_S72
VR_kl_S73
VR_kl_S74
VR_kl_S75
VR_kl_S76
VR_kl_S77
VR_kl_S78
VR_kl_S79
VR_kl_S80
VR_kl_S81
VR_kl_S82
VR_kl_S83
VR_kl_S84
VR_kl_S85
VR_kl_S86
VR_kl_S87
VR_kl_S88
VR_kl_S89
VR_kl_S90
VR_kl_S91
VR_kl_S92
VR_kl_S93

VR_kl_S94
VR_kl_S95
VR_kl_S96
VR_kl_S97
VR_kl_S98
VR_kl_S99
VR_kl_T01
VR_kl_T02
VR_kl_T03
VR_kl_T04
VR_kl_T05
VR_kl_T06
VR_kl_T07
VR_kl_T08
VR_kl_T10
VR_kl_T11
VR_kl_T15
VR_kl_T26
VR_kl_T27
VR_kl_T28
VR_kl_T29
VR_kl_T70
VR_kl_T71
VR_kl_T72
VR_kl_T73
VR_kl_T78
VR_kl_T80
VR_kl_T81
VR_kl_T82
VR_kl_T83
VR_kl_T85
VR_kl_T86
VR_kl_T87
VR_kl_T88
VR_kl_T90
VR_kl_T91
VR_kl_T92
VR_kl_T93
VR_kl_T99
VR_kl_U01
VR_kl_U02
VR_kl_U04
VR_kl_U05
VR_kl_U06
VR_kl_U07
VR_kl_U13
VR_kl_U14
VR_kl_U26
VR_kl_U27
VR_kl_U28
VR_kl_U29
VR_kl_U70

VR_kl_U71
VR_kl_U72
VR_kl_U75
VR_kl_U76
VR_kl_U77
VR_kl_U78
VR_kl_U79
VR_kl_U80
VR_kl_U85
VR_kl_U88
VR_kl_U90
VR_kl_U95
VR_kl_U98
VR_kl_U99
VR_kl_W01
VR_kl_W02
VR_kl_W03
VR_kl_W05
VR_kl_W10
VR_kl_W11
VR_kl_W12
VR_kl_W13
VR_kl_W14
VR_kl_W15
VR_kl_W17
VR_kl_W18
VR_kl_W19
VR_kl_W20
VR_kl_W27
VR_kl_W28
VR_kl_W29
VR_kl_W70
VR_kl_W71
VR_kl_W72
VR_kl_W73
VR_kl_W75
VR_kl_W76
VR_kl_W77
VR_kl_W78
VR_kl_W79
VR_kl_W80
VR_kl_W81
VR_kl_W82
VR_kl_W83
VR_kl_W84
VR_kl_W90
VR_kl_W91
VR_kl_W92
VR_kl_W93
VR_kl_W94
VR_kl_W95
VR_kl_W96

VR_kl_W99
VR_kl_X01
VR_kl_X02
VR_kl_X03
VR_kl_X04
VR_kl_X05
VR_kl_X06
VR_kl_X07
VR_kl_X08
VR_kl_X09
VR_kl_X10
VR_kl_X11
VR_kl_X12
VR_kl_X13
VR_kl_X14
VR_kl_X15
VR_kl_X16
VR_kl_X17
VR_kl_X18
VR_kl_X19
VR_kl_X20
VR_kl_X21
VR_kl_X23
VR_kl_X24
VR_kl_X25
VR_kl_X26
VR_kl_X27
VR_kl_X28
VR_kl_X29
VR_kl_X70
VR_kl_X71
VR_kl_X72
VR_kl_X73
VR_kl_X74
VR_kl_X75
VR_kl_X76
VR_kl_X77
VR_kl_X78
VR_kl_X79
VR_kl_X80
VR_kl_X81
VR_kl_X82
VR_kl_X83
VR_kl_X84
VR_kl_X85
VR_kl_X86
VR_kl_X87
VR_kl_X88
VR_kl_X89
VR_kl_X90
VR_kl_X91
VR_kl_X99

VR_kl_Y01
VR_kl_Y02
VR_kl_Y03
VR_kl_Y04
VR_kl_Y05
VR_kl_Y06
VR_kl_Y07
VR_kl_Y08
VR_kl_Y10
VR_kl_Y13
VR_kl_Y14
VR_kl_Y16
VR_kl_Y24
VR_kl_Y25
VR_kl_Y26
VR_kl_Y27
VR_kl_Y28
VR_kl_Y29
VR_kl_Y70
VR_kl_Y71
VR_kl_Y72
VR_kl_Y73
VR_kl_Y74
VR_kl_Y75
VR_kl_Y76
VR_kl_Y77
VR_kl_Y78
VR_kl_Y79
VR_kl_Y80
VR_kl_Y81
VR_kl_Y82
VR_kl_Y83
VR_kl_Y84
VR_kl_Y85
VR_kl_Y86
VR_kl_Y99
VR_kl_Z01
VR_kl_Z02
VR_kl_Z03
VR_kl_Z04
VR_kl_Z05
VR_kl_Z06
VR_kl_Z07
VR_kl_Z08
VR_kl_Z09
VR_kl_Z10
VR_kl_Z11
VR_kl_Z12
VR_kl_Z13
VR_kl_Z14
VR_kl_Z15
VR_kl_Z16

VR_kl_Z18
VR_kl_Z19
VR_kl_Z20
VR_kl_Z21
VR_kl_Z22
VR_kl_Z23
VR_kl_Z24
VR_kl_Z25
VR_kl_Z27
VR_kl_Z28
VR_kl_Z29

Code group
klacht of diagnose waarover de vraag gesteld wordt
Gegeneraliseerde pijn
Koude rillingen
Koorts
Moeheid/zwakte
Algehele achteruitgang
Flauwvallen/syncope
Coma
Zwelling [ex. K07]
Transpiratieproblemen
Bloeding
Allergie/allergische reactie
Bezorgdheid over (bij)werking geneesmiddel
Koliek bij zuigeling
Overmatig huilende zuigeling
Prikkelbare/drukke zuigeling
Algemene symptomen/klachten zuigeling
Gesprek levenseinde/behandelwensen
Angst voor de dood
Angst voor kanker
Angst voor andere ziekte
Functiebeperking/handicap
Andere algemene symptomen/klachten
Andere reden voor contact
Gegeneraliseerde tuberculose [ex. R70]
Mazelen
Waterpokken
Malaria
Rode hond
Mononucleosis infectiosa
Andere virusziekte met exantheem
Andere virusziekte(n)
Andere infectieziekte(n)
Maligniteit met onbekende primaire lokalisatie
Trauma/letsel
Multiple traumata/inwendig letsels
Laat gevolg van letsel
Geneesmiddelintoxicatie
Geneesmiddelbijwerking
Intoxicatie andere chemische stof
Complicatie(s) medische behandeling
Schadelijk gevolg fysische factor [ex. H85]
Aanwezigheid/gevolg prothese
Multiple aangeboren afwijkingen
Afwijkende uitslag(en) onderzoek
Toxoplasmose
Dysmatuur/prematuur/immatuur levendgeborene
Perinatale morbiditeit
Perinatale mortaliteit
Dood/overlijden [ex. A95]
Geen ziekte

Andere gegeneraliseerde/niet gespecificeerde ziekte(n)
Vergrote lymfeklier(en)
Andere symptomen/klachten lymfeklieren
Symptomen/klachten bloed/bloedvormende organen
Angst voor AIDS
Angst voor kanker bloed/lymfestelsel
Angst voor andere ziekte bloed/lymfestelsel
Functiebeperking/handicap bloed/lymfestelsel
Andere symptomen/klachten bloed/lymfestelsel
Acute lymphadenitis
Chronische/niet-gespecificeerde lymphadenitis
Ziekte van Hodgkin
Leukemie
Andere maligniteit bloed/lymfestelsel
Benigne/niet-gespecificeerd neoplasma bloed/lymfestelsel
Miltruptuur
Ander letsel bloed/lymfestelsel
Erfelijke hemolytische anemie
Andere aangeboren afwijking bloed/lymfestel
IJzergebrekanemie
Pernicieuze/foliumzuurdeficiëntie-anemie
Andere/niet-gespecificeerde anemie
Purpura/stollingsstoornis/afwijkende trombocyten
Afwijking leukocyten
Onverklaarde afwijking bloedonderzoek
Andere hematologische afwijking(en)
Splenomegalie
HIV-infectie (AIDS/ARC)
Andere ziekte(n) bloed/lymfestelsel/milt
Gegeneraliseerde buikpijn/buikkrampen
Maagpijn
Zuurbranden
Pijn anus/rectum
Perianale jeuk
Andere gelokaliseerde buikpijn
Flatulentie/meteorisme/boeren
Misselijkheid
Braken
Diarree
Obstipatie
Geelzucht
Haematemesis
Melaena
Rectaal bloedverlies
Incontinentie voor ontlasting
Verandering ontlasting/defecatiepatroon
Symptomen/klachten tanden/tandvlees
Symptomen/klachten mond/tong/lippen/speekselklieren
Slikproblemen
Wormen/andere parasieten
Zwelling in de buik
Verandering omvang/uitzetting buik

Angst voor kanker spijsverteringsorganen
Angst voor andere ziekte spijsverteringsorganen
Functiebeperking/handicap spijsverteringsorganen
Andere symptomen/klachten spijsverteringsorganen
Infectieuze diarree, dysenterie
Bof
Virus hepatitis
Veronderstelde gastro-intestinale infectie
Maligniteit maag
Maligniteit colon/rectum
Maligniteit pancreas
Andere/niet-gespecif.maligniteit spijsverteringsorganen
Benigne neoplasma spijsverteringsorganen
Corpus alienum via mond/anus
Ander letsel spijsverteringsorganen
Aangeboren afwijking(en) spijsverteringsorganen
Ziekte tanden/tandvlees
Ziekte mond/tong/lippen/speekselklieren
Ziekte oesofagus
Ulcus duodeni
Ander ulcus pepticum
Stoornis maagfunctie
Appendicitis
Hernia inguinalis
Hernia diafragmatica/hiatus
Andere hernia abdominalis
Diverticulose/diverticulitis
Prikkelbare darm syndroom
Colitis ulcerosa/chronische enteritis (regionalis)
Fissura ani/perianaal abces
Hepatomegalie
Cirrose/andere leverziekte
Cholecystitis/cholelithiasis
Andere ziekte(n) spijsverteringsorganen
Pijn oog
Rood oog
Afscheiding uit oog
Mouches volantes/flitsen/flikkeringen
Andere visussymptomen/-klachten [ex. F94]
Afwijkend gevoel aan oog
Afwijkende oogbewegingen
Afwijkend aspect oog
Symptomen/klachten oogleden
Symptomen/klachten van bril
Symptomen/klachten van contactlens
Angst voor ziekte oog
Functiebeperking/handicap oog/adnexen
Andere symptomen/klachten oog/adnexen
Infectieuze conjunctivitis
Blepharitis/hordeolum/chalazion
Andere infectie/ontsteking oog/adnexen [ex. F85,F86]
Neoplasma oog/adnexen

Kneuzing/bloeding oog/adnexen
Corpus alienum oog
Ander letsel oog/adnexen
Stenose traankanaal zuigeling
Andere aangeboren afwijking(en) oog/adnexen
Netvliesloslating
Retinopathie
Maculadegeneratie
Ulcus corneae
Trachoom
Refractie afwijking(en)
Cataract/staar
Glaucoom/verhoogde oogdruk
Blindheid (elke graad/vorm)
Strabismus/scheelzien
Andere ziekte(n) oog/adnexen
Oorpijn
Gehoorklachten [ex. H84,H85,H86]
Oorsuizen/tinnitus
Afscheiding uit oor
Bloed in/uit oor
Verstopt gevoel oor
Ontevreden/bezorgd over aspect oor
Angst voor ziekte oor
Functiebeperking/handicap oor
Andere symptomen/klachten oor
Otitis externa
Otitis media acuta/myringitis
Otitis media met effusie
Tubair catarre/tubastenose
Chronische otitis media/andere infectie oor
Neoplasma oor
Corpus alienum in oor
Perforatie trommelvlies [ex. H71]
Oppervlakkig letsel oor
Ander letsel oor
Aangeboren afwijking(en) oor
Overmatig cerumen
Vertigosyndroom/labyrinthitis [ex. N17]
Otosclerose
Presbyacusis
Akoestisch letsel/lawaaidoofheid
Doofheid/slechthorendheid
Andere ziekte(n) oor
Pijn toegeschreven aan hart
Druk/beklemming toegeschreven aan hart [ex. R02]
Andere pijn toegeschreven aan hartvaatstelsel
Hartkloppingen/bewust van hartslag
Andere afwijkende/onregelmatige hartslag
Opgezette aderen
Gezwollen enkels/enkeloedeem
Angst voor hartaanval

Angst voor hoge bloeddruk
Angst voor andere ziekte hartvaatstelsel
Functiebeperking/handicap hartvaatstelsel
Andere symptomen/klachten hartvaatstelsel
Infectieziekte hartvaatstelsel
Acuut reuma/reumatische hartziekte
Neoplasma hartvaatstelsel
Aangeboren afwijking(en) hartvaatstelsel
Angina pectoris
Acuut myocardinfarct
Andere/chronische ischemische hartziekte
Decompensatio cordis
Boezemfibrilleren/-fladderen
Paroxysmale tachycardie
Ectopische slagen/extrasystolen
Hartgeruis
Cor pulmonale
Niet-reumatische klepaandoening
Andere hartziekte(n)
Verhoogde bloeddruk [ex. K86,K87]
Essentiële hypertensie zonder orgaanbeschadiging
Hypertensie met orgaanbeschadiging/secundaire hypertensie
Orthostatische hypotensie
Passagère cerebrale ischemie/TIA
Cerebrovasculair accident (CVA)
Atherosclerose [ex. K76,K90]
Andere ziekte(n) perifere arteriën
Longembolie/longinfarct
Thrombophlebitis/flebotrombose
Varices benen [ex. S97]
Hemorroïden
Andere ziekte(n) hartvaatstelsel
Nek symptomen/klachten [ex. N01]
Rug symptomen/klachten
Lage-rugpijn zonder uitstraling [ex. L86]
Borstkas symptomen/klachten
Flank symptomen/klachten
Oksel symptomen/klachten
Kaak(gewricht) symptomen/klachten
Schouder symptomen/klachten
Arm symptomen/klachten
Elleboog symptomen/klachten
Pols symptomen/klachten
Hand/vinger symptomen/klachten
Heup symptomen/klachten
Been/dijbeen symptomen/klachten
Knie symptomen/klachten
Enkel symptomen/klachten
Voet/teen symptomen/klachten
Spierpijn
Symptomen meerdere/niet-gespecificeerde spieren
Symptomen meerdere/niet-gespecificeerde gewrichten

Angst voor kanker bewegingsapparaat
Angst voor andere ziekte bewegingsapparaat
Functiebeperking/handicap bewegingsapparaat
Andere/meerdere symptomen/klachten bewegingsapparaat
Infectieziekte bewegingsapparaat
Neoplasma bewegingsapparaat
Fractuur radius/ulna
Fractuur tibia/fibula
Fractuur hand/voet zwelling
Fractuur femur
Andere fractuur
Verstuiking/distorsie enkel
Verstuiking/distorsie knie
Andere verstuiking/distorsie
Luxatie/subluxatie
Ander letsel bewegingsapparaat
Aangeboren afwijking(en) bewegingsapparaat
Syndroom cervicale wervelkolom
Artrose/spondylose wervelkolom
Verworven afwijking(en) wervelkolom
Lage-rugpijn met uitstraling
Ganglion gewricht/pees
Reumatoïde artritis/verwante aandoening(en)
Coxartrose
Gonartrose
Andere artrose/verwante aandoening(en)
Schoudersyndroom/PHS
Epicondylitis lateralis
Osgood-Schlatter/andere osteochondropathie
Osteoporose
Acuut letsel meniscus/kniebanden
Chronisch inwendig trauma knie
Verworven afwijking(en) extremiteiten
Andere ziekte(n) bewegingsapparaat
Hoofdpijn [ex. N02,N89,R09]
Spanningshoofdpijn
Aangezichtspijn
Restless legs
Tintelen vingers/voeten/tenen
Andere sensibiliteitstoornis/onwillekeurige bewegingen
Convulsies/stuipen (inclusief koorts-)
Andere afwijking(en) reuk/smaak
Vertigo/duizeligheid [ex. H82]
Verlamming/krachtverlies [ex. A04]
Spraak-/fonatiestoornis
Angst voor kanker zenuwstelsel
Angst voor andere ziekte zenuwstelsel
Functiebeperking/handicap zenuwstelsel
Andere symptomen/klachten zenuwstelsel
Poliomyelitis/andere enterovirus infectie
Meningitis/encephalitis
Tetanus

Andere infectieziekte(n) zenuwstelsel
Maligniteit zenuwstelsel
Benigne neoplasma zenuwstelsel
Niet-gespecificeerd neoplasma zenuwstelsel
Hersenschudding/hoofdtrauma
Ander letsel hoofd [ex. L76]
Ander letsel zenuwstelsel
Aangeboren afwijking(en) zenuwstelsel
Multiple sclerose
Parkinsonisme, ziekte van Parkinson
Epilepsie (alle vormen)
Migraine
Cluster headache
Facialis parese/Bell's palsy
Trigeminus neuralgie
Carpale tunnelsyndroom
Andere perifere neuritis/neuropathie
Andere ziekte(n) zenuwstelsel
Angstig/nerveus/gespannen gevoel
Crisis/voorbijgaande stressreactie
Down/depressief gevoel
Prikkelbaar/boos gevoel/gedrag
Zich oud voelen/gedragen
Slapeloosheid/andere slaapstoornis
Libido verlies/vermindering
Seksuele bevrediging verlies/vermindering
Bezorgdheid over seksuele voorkeur
Stamelen/stotteren/tics
Eetprobleem(en) bij kind
Enuresis [ex. U04]
Encopresis
Chronisch alcoholmisbruik
Acuut alcohol misbruik/intoxicatie
Tabaksmisbruik
Geneesmiddelmisbruik
Drugsmisbruik
Geheugen-/concentratie-/oriëntatiestoornissen
Overactief (kind)/hyperkinetisch syndroom
Andere zorgen gedrag kind
Andere zorgen gedrag adolescent
Specifiek leerprobleem
Levensfaseprobleem volwassene
Angst voor psychische ziekte
Functiebeperking/handicap psychische ziekte
Andere psychische symptomen/klachten
Seniele dementie/Alzheimer
Andere organische psychose(n)
Schizofrenie
Affectieve psychose
Angststoornis/angsttoestand
Somatisatiestoornis
Depressie

Suïcidepoging
Neurasthenie/surmenage
Andere neurose
Persoonlijkheids-/karakterstoornis
Mentale retardatie/intellectuele achterstand
Andere/niet gespecificeerde psychose(n)
Andere psychische stoornissen
Pijn toegeschreven aan luchtwegen [ex. R09]
Dyspnoe/benauwdheid toegeschreven aan luchtwegen [ex. K02]
Piepende ademhaling
Andere problemen ademhaling
Hoesten
Epistaxis/neusbloeding
Niezen/neusverstopping/loopneus
Andere symptomen/klachten neus
Symptomen/klachten sinussen (inclusief pijn)
Symptomen/klachten keel
Symptomen/klachten tonsillen
Symptomen/klachten stem
Haemoptoë
Abnormaal sputum/slijm
Angst voor kanker luchtwegen
Angst voor andere ziekte luchtwegen
Functiebeperking/handicap luchtwegen
Andere symptomen/klachten luchtwegen
Tuberculose luchtwegen [ex. A70]
Kinkhoest
Streptokokken-angina/roodvonk
Furunkel/abces neus
Acute infectie bovenste luchtwegen
Acute/chronische rinosinusitis
Acute tonsillitis/peritonsillair abces
Acute laryngitis/tracheïtis
Acute bronchitis/bronchiolitis
Influenza [ex. R81]
Pneumonie
Pleuritis alle vormen [ex. R70]
Andere infectie(s) luchtwegen
Maligniteit bronchus/long
Andere maligniteit luchtwegen
Benigne neoplasma luchtwegen
Corpus alienum neus/larynx/bronchiën
Ander letsel luchtwegen
Aangeboren afwijking(en) luchtwegen
Hypertrofie/chronische infectie tonsillen/adenoïd
Chronische bronchitis/bronchiëctasieën
Pleuravocht
Emfyseem/COPD
Astma
Hooikoorts/allergische rinitis
Hyperventilatie
Andere ziekte(n) luchtwegen

Pijn/gevoeligheid huid
Pruritus/jeuk [ex. D05,X16]
Wratten
Lokale zwelling/papel/knobbel huid/subcutis
Multiple zwellingen/papels/knobbels huid/subcutis
Lokale roodheid/erytheem huid
Gegeneraliseerde roodheid/erytheem huid
Andere verandering(en) in kleur huid
Lokale infectie vinger/teen/paronychia
Furunkel/karbunkel/cellulitis lokaal
Andere lokale infectie(s) huid/subcutis
Beet/steek insekt
Beet mens/dier
Brandwond/verbranding huid (elke graad)
Corpus alienum huid/subcutis
Buil/kneuzing/contusie intacte huid
Schaafwond/schram/blaar
Scheurwond/snijwond
Ander letsel van de huid/subcutis
Likdoorn(s)/eeltknobbel(s)
Andere symptomen/klachten aspect huid
Symptomen/klachten nagels
Haaruitval/alopecia
Andere symptomen/klachten haar
Angst voor kanker huid/subcutis
Angst voor andere ziekte huid/subcutis
Functiebeperking/handicap huid/subcutis
Andere symptomen/klachten huid/subcutis
Herpes zoster
Herpes simplex [ex. F85,X90,Y72]
Scabies/andere aandoening door mijten
Pediculosis/andere huidinfestatie
Dermatomycose(n)
Moniliasis/candidiasis [ex. X72,Y75]
Andere infectie huid/subcutis
Maligniteit huid/subcutis
Lipoom
Ander benigne neoplasma huid/subcutis
Ander/niet-gespecificeerd neoplasma huid/subcutis
Hemangioom/lymfangioom
Naevus/moedervlek
Andere aangeboren afwijking(en) huid/subcutis
Impetigo/impetiginisatie
Pilonidaal cyste/fistel
Seborroïsch eczeem/roos
Constitutioneel eczeem
Contact eczeem/ander eczeem
Luiereczeem
Pityriasis rosea
Psoriasis
Ziekte(n) zweetklieren
Atheroomcyste/epitheelcyste

Unguis incarnatus/andere nagelaandoening
Mollusca contagiosa
Acne
Ulcus cruris/decubitus/chronisch ulcus
Urticaria
Andere ziekte(n) huid/subcutis
Overmatige dorst
Overmatige eetlust
Verminderde eetlust
Voedingsprobleem zuigeling/kind [ex. P11]
Voedingsprobleem volwassene [ex. T06]
Anorexia nervosa/boulimie
Gewichtstoename
Gewichtsverlies
Achterblijven verwachte fysiologische ontwikkeling
Dehydratie
Knobbel/zwelling schildklier
Angst voor kanker endocriene klieren
Angst andere ziekte endocriene klieren/metabolisme/voeding
Funct.beperking/handicap endocr. klieren/metabolisme/voeding
And. sympt./klacht. endocr. klieren/metabolisme/voeding
Infectie endocriene klier(en)
Maligniteit schildklier
Benigne neoplasma schildklier
Ander/niet-gespecificeerd neoplasma endocriene klieren
Persisterende ductus thyreoglossus/cyste
Andere aangeboren afwijking endocriene klieren/metabolisme
Struma/noduli [ex. T85,T86]
Adipositas
Overgewicht
Hyperthyreoïdie/thyreotoxicose
Hypothyreoïdie/myxoedeem
Hypoglykemie
Renale glucosurie
Diabetes mellitus
Vitamine-/voedingsdeficiëntie(s)
Jicht
Vetstofwisselingsstoornis(sen)
Andere ziekte(n) endocriene klieren/metabolisme/voeding
Pijnlijke mictie
Frequente mictie/aandrang
Urine-incontinentie [ex. P12]
Ander mictieprobleem
Hematurie
Andere symptomen/klachten urine
Andere symptomen/klachten blaas
Symptomen/klachten nieren
Angst voor kanker urinewegen
Angst voor andere ziekte urinewegen
Functiebeperking/handicap urinewegen
Andere symptomen/klachten urinewegen
Acute pyelonephritis/pyelitis
