# Supplementary figures and images for: Evaluation of the First Year(s) of Physicians Collaboration on an Interdisciplinary Electronic Consultation Platform in the Netherlands: Mixed Methods Observational Study
Source: JMIR Hum Factors. 2022 Apr 1;9(2):e33630. doi: 10.2196/33630 (PMC9015779; doi:10.2196/33630)

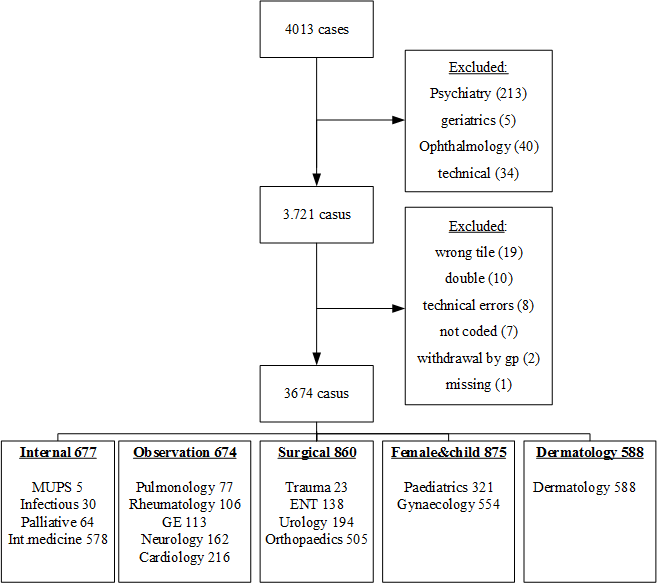

Supplement: Multimedia Appendix 2 [file humanfactors_v9i2e33630_app2.png]

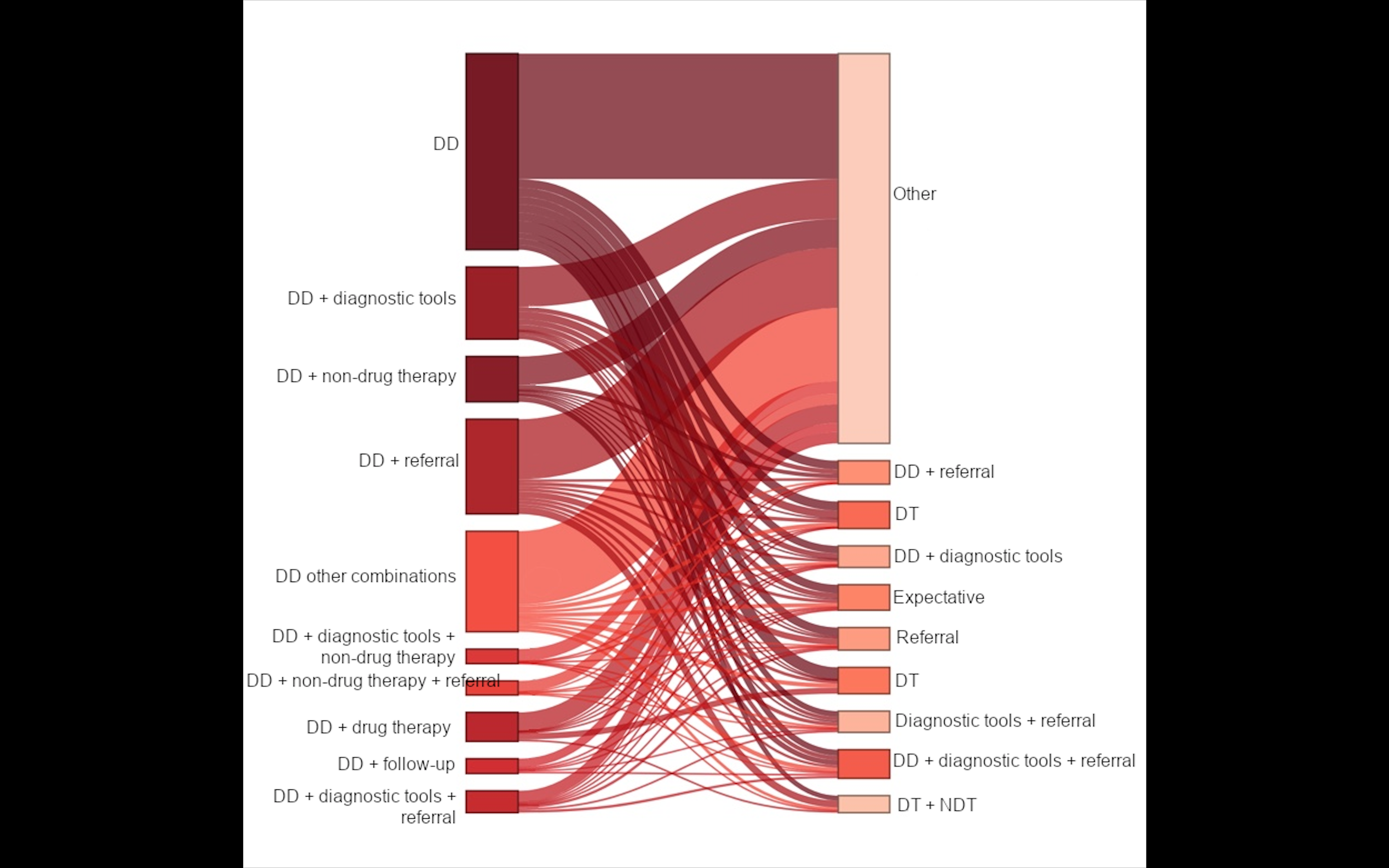

Supplement: Multimedia Appendix 6 [file humanfactors_v9i2e33630_app6.png]

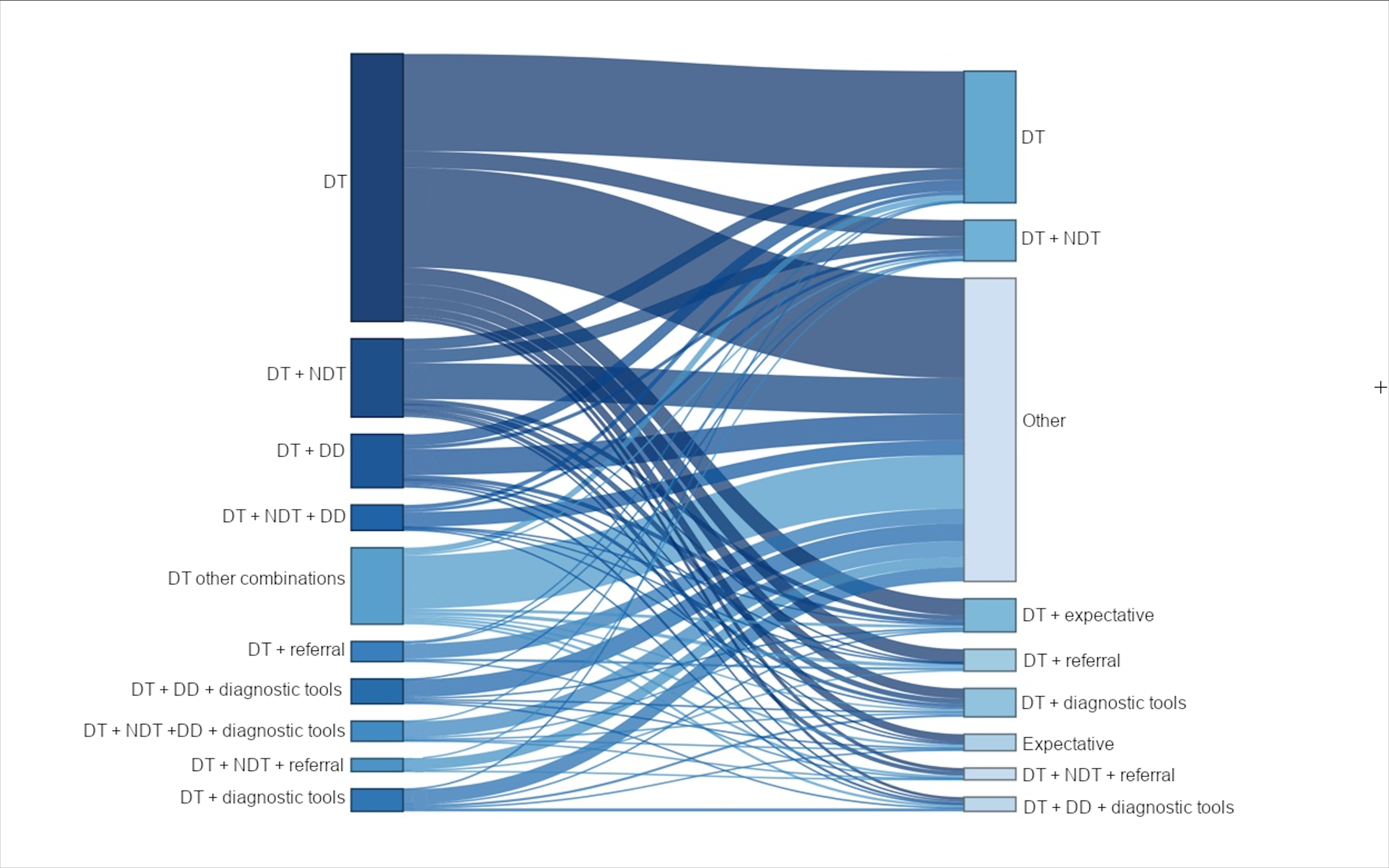

Supplement: Multimedia Appendix 7 [file humanfactors_v9i2e33630_app7.png]

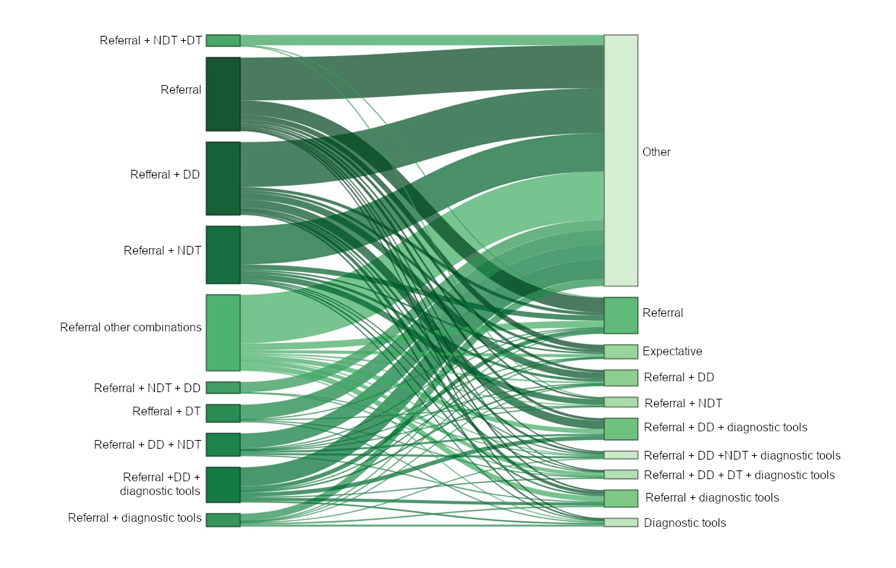

Supplement: Multimedia Appendix 8 [file humanfactors_v9i2e33630_app8.png]
