# Supplementary material for: Evaluation of the First Year(s) of Physicians Collaboration on an Interdisciplinary Electronic Consultation Platform in the Netherlands: Mixed Methods Observational Study
Source: JMIR Hum Factors. 2022 Apr 1;9(2):e33630. doi: 10.2196/33630 (PMC9015779; doi:10.2196/33630)
Supplement: Multimedia Appendix 3 [file humanfactors_v9i2e33630_app3.doc]

|  | **All** | **Internal** | **Observation** | **Surgical** | **Female/Child** | **Dermatology** |
| --- | --- | --- | --- | --- | --- | --- |
| **All cases (n)** | 3,674 | 677 | 674 | 860 | 875 | 588 |
| **Cases with answer**, n | 3,645 | 673 | 665 | 855 | 872 | 580 |
| **Time to first answer**, minutes, median [IQR] | 76 [17–20] | 90 [25–284] | 48 [15–209] | 44 [12–178] | 71 [13–417] | 252 [66–799] |
| **Cases with information**, n | 3508 | *638* | 626 | 834 | 833 | 577 |
| **Gender**,% |  |  |  |  |  |  |
| Female  Male  Missing | 54.6  36.4  9.1 | 59.4  36.7  3.9 | 53.8  42.5  3.7 | 43.5  52.3  4.2 | 63  14.5  22.5 | 54.2  38.3  7.5 |
| **Age (y)**, Mean ± SD  Range  Numbers  Missing | 39.9 ± 24.6  0–101  3298  210 | 53.5 ± 20.7  13–95  575  63 | 50.9 ± 21.1  3–101  626  0 | 43.7 ± 23.8  0–97  808  26 | 23.7 ± 19.2  0–89  781  52 | 38.0 ± 25.1  0–96  508  69 |

Abbreviations: GP, general practitioner; SD, standard deviation.
