# Supplementary material for: Evaluation of the First Year(s) of Physicians Collaboration on an Interdisciplinary Electronic Consultation Platform in the Netherlands: Mixed Methods Observational Study
Source: JMIR Hum Factors. 2022 Apr 1;9(2):e33630. doi: 10.2196/33630 (PMC9015779; doi:10.2196/33630)
Supplement: Multimedia Appendix 4 [file humanfactors_v9i2e33630_app4.doc]

| **Category** | **Total** | **A** | **B** | **C** | **D** | **E** |
| --- | --- | --- | --- | --- | --- | --- |
| Total questions, n | 5825 | 1045 | 1062 | 924 | 1504 | 1290 |
| **General**,% | *14.2* | *31.5* | *20.6* | *2.7* | *13.6* | *4.4* |
| **Immune/blood**,% | *6.0* | *26.6* | *2.8* | *0.5* | *1.4* | *1.2* |
| **Digestive**,% | *9.6* | *9.5* | *15.4* | *4.5* | *14.2* | *3.4* |
| **Eye**,% | *1.0* | *1.0* | *2.1* | *0.1* | *0.8* | *1.2* |
| **Ear**,% | *1.6* | *0.7* | *0.7* | *4.8* | *0.9* | *0* |
| **Cardiovascular**,% | *6.7* | *8.6* | *24.9* | *0.1* | *1.4* | *1.2* |
| **Musculoskeletal**,% | *20.8* | *11.2* | *22.0* | *56* | *5.3* | *2.2* |
| **Neurological**,% | *5.2* | *5.8* | *15.9* | *1.7* | *2.6* | *1.2* |
| **Psychiatry**,% | *4.0* | *3.0* | *5.6* | *2.6* | *7.1* | *0.7* |
| **Respiratory**,% | *4.7* | *6.6* | *9.3* | *4.0* | *2.5* | *1.5* |
| **Skin**,% | *20.6* | *8.1* | *2.8* | *2.9* | *11* | *95.6* |
| **Endo/nutrition**,% | *6* | *16.4* | *4.7* | *0.9* | *7.8* | *0.2* |
| **Urology**,% | *3.8* | *4.1* | *0.9* | *10.6* | *1.4* | *0.5* |
| **Pregnancy/family**,% | *7.9* | *1.9* | *0.3* | *0.1* | *31.0* | *0.5* |
| **Female genital**,% | *10.9* | *2.5* | *0.4* | *0.6* | *41.7* | *1.9* |
| **Male genital**,% | *2.8* | *1.0* | *0* | *9.0* | *0.9* | *1.9* |
| **Social**,% | *0.1* | *0.1* | *0.1* | *0.2* | *0.2* | *0.2* |

Category Names

A: internal

B: observation

C: surgical

D: female and child

E: dermatology
