# Supplementary material for: Evaluation of the First Year(s) of Physicians Collaboration on an Interdisciplinary Electronic Consultation Platform in the Netherlands: Mixed Methods Observational Study
Source: JMIR Hum Factors. 2022 Apr 1;9(2):e33630. doi: 10.2196/33630 (PMC9015779; doi:10.2196/33630)
Supplement: Multimedia Appendix 5 [file humanfactors_v9i2e33630_app5.doc]

|  | **All** | **Internal** | **Observation** | **Surgical** | **Female/Child** | **Derma** |
| --- | --- | --- | --- | --- | --- | --- |
| **All cases**, n | 3,674 | 677 | 674 | 860 | 875 | 588 |
| **Different questions**, n | 6,691 | 1,334 | 1,280 | 1,505 | 1,507 | 1,065 |
| **Question/case**, mean | 1.82 | 1.97 | 1.90 | 1.75 | 1.72 | 1.81 |
| **Question category**,% |  |  |  |  |  |  |
| (Differential) diagnosis | 50.9 | 52.9 | 53.6 | 43.5 | 40.3 | 72.1 |
| Nondrug treatment | 33.2 | 31.0 | 21.2 | 50.1 | 14.3 | 52.6 |
| Drug treatment | 27.6 | 33.8 | 28.5 | 13.5 | 43.2 | 16.8 |
| Referral | 20.5 | 18.9 | 24.8 | 33.8 | 15.8 | 5.3 |
| Diagnostic tools | 19.9 | 29.5 | 30.4 | 7.7 | 24.1 | 8.5 |
| Other | 14.5 | 8.6 | 11.7 | 14.4 | 17.8 | 19.6 |
| Follow-up | 6.2 | 12.6 | 9.9 | 4.1 | 4.5 | 0 |
| Natural course | 4.8 | 4.0 | 2.7 | 4.9 | 7.4 | 4.4 |
| Non patient-specific | 4.5 | 5.8 | 7.1 | 3.0 | 4.8 | 1.9 |
| **Different answers, n** | 10,922 | 1,960 | 2,225 | 2,889 | 2,375 | 1,473 |
| **Answer/case**, mean | 3.03 | 2.96 | 3.34 | 3.39 | 2.77 | 2.60 |
| **Answer category**,% |  |  |  |  |  |  |
| (differential) diagnosis | 66.2 | 59.8 | 77.7 | 68.7 | 50.1 | 81 |
| Diagnostic tool | 41.6 | 52.3 | 51.8 | 44.0 | 33.1 | 26.8 |
| Drug treatment | 39.8 | 38.4 | 44.2 | 30.2 | 45.1 | 42.5 |
| Referral | 38.3 | 30.3 | 47.6 | 52.6 | 35.7 | 19.7 |
| Background information | 37.2 | 45.9 | 34.0 | 34.6 | 40.2 | 28.4 |
| Nondrug treatment | 28.2 | 24.4 | 24.9 | 42.6 | 15.7 | 34.2 |
| Expectative | 26.5 | 26.9 | 26.0 | 29.5 | 33.3 | 12.4 |
| GP to follow-up | 11.4 | 8.1 | 16.9 | 14.0 | 11.4 | 4.8 |
| Paramedical treatment | 7.3 | 1.9 | 6.8 | 17.4 | 5.9 | 1.0 |
| Other | 0.8 | 1.5 | 0.1 | 1.4 | 0.9 | 0 |

Abbreviations: GP, general practitioner;
